# Resting Brain Fluctuations Are Intrinsically Coupled to Visual Response Dynamics

**DOI:** 10.1093/cercor/bhaa305

**Published:** 2020-10-27

**Authors:** Michaël E Belloy, Jacob Billings, Anzar Abbas, Amrit Kashyap, Wen-Ju Pan, Rukun Hinz, Verdi Vanreusel, Johan Van Audekerke, Annemie Van der Linden, Shella D Keilholz, Marleen Verhoye, Georgios A Keliris

**Affiliations:** Department of Pharmaceutical, Veterinary and Biomedical Sciences, University of Antwerp, 2610 Antwerp, Belgium; Department of Biomedical Engineering, Emory University and Georgia Institute of Technology, Atlanta, GA 30322, USA; Department of Neuroscience, Emory University, Atlanta, GA 30322, USA; Department of Neuroscience, Emory University, Atlanta, GA 30322, USA; Department of Biomedical Engineering, Emory University and Georgia Institute of Technology, Atlanta, GA 30322, USA; Department of Biomedical Engineering, Emory University and Georgia Institute of Technology, Atlanta, GA 30322, USA; Department of Pharmaceutical, Veterinary and Biomedical Sciences, University of Antwerp, 2610 Antwerp, Belgium; Department of Pharmaceutical, Veterinary and Biomedical Sciences, University of Antwerp, 2610 Antwerp, Belgium; Department of Pharmaceutical, Veterinary and Biomedical Sciences, University of Antwerp, 2610 Antwerp, Belgium; Department of Pharmaceutical, Veterinary and Biomedical Sciences, University of Antwerp, 2610 Antwerp, Belgium; Department of Neuroscience, Emory University, Atlanta, GA 30322, USA; Department of Pharmaceutical, Veterinary and Biomedical Sciences, University of Antwerp, 2610 Antwerp, Belgium; Department of Pharmaceutical, Veterinary and Biomedical Sciences, University of Antwerp, 2610 Antwerp, Belgium

**Keywords:** brain state, default mode (DMN) and task-positive network (TPN), functional MRI, neuromodulation, visual stimulation

## Abstract

How do intrinsic brain dynamics interact with processing of external sensory stimuli? We sought new insights using functional magnetic resonance imaging to track spatiotemporal activity patterns at the whole brain level in lightly anesthetized mice, during both resting conditions and visual stimulation trials. Our results provide evidence that quasiperiodic patterns (QPPs) are the most prominent component of mouse resting brain dynamics. These QPPs captured the temporal alignment of anticorrelation between the default mode (DMN)- and task-positive (TPN)-like networks, with global brain fluctuations, and activity in neuromodulatory nuclei of the reticular formation. Specifically, the phase of QPPs prior to stimulation could significantly stratify subsequent visual response magnitude, suggesting QPPs relate to brain state fluctuations. This is the first observation in mice that dynamics of the DMN- and TPN-like networks, and particularly their anticorrelation, capture a brain state dynamic that affects sensory processing. Interestingly, QPPs also displayed transient onset response properties during visual stimulation, which covaried with deactivations in the reticular formation. We conclude that QPPs appear to capture a brain state fluctuation that may be orchestrated through neuromodulation. Our findings provide new frontiers to understand the neural processes that shape functional brain states and modulate sensory input processing.

## Introduction

Resting-state functional magnetic resonance imaging (rsfMRI) and task-evoked fMRI are powerful complementary techniques to study brain function (Fox and Raichle 2007; Bandettini 2012). The first investigates the intrinsically highly active nature of the brain, while the second studies the brain’s reflexive properties and less so considers the “background” intrinsic fluctuations that are averaged out across trials (Raichle 2010). Recent studies support the view that intrinsic blood oxygen level-dependent (BOLD) fluctuations across individual trials affect sensory responses and behavioral performance (Boly et al. 2007; Fox et al. 2006, 2007; He 2013; Shimaoka et al. 2019). Yet, it remains unclear which specific regional or brain-wide neural mechanisms underlie this interaction.

Answers may come from emerging tools in the field of time-resolved rsfMRI, which attempts to identify the dynamic interaction of brain networks during the resting state (Deco et al. 2011; Allen et al. 2014; Keilholz 2014). Brain states or cognitive fluctuations may be identified and their role in task performance evaluated (Gonzalez-Castillo et al. 2015; Keilholz et al. 2017; Kucyi et al. 2018). Changes in vigilance or attention may also be identified and appear difficult to dissociate from cognitive brain states (Tagliazucchi and Laufs 2014; Wang et al. 2016; Laumann et al. 2017; Allen et al. 2018; Hinz et al. 2019).

To improve understanding of brain state dynamics and associated properties, new insights may come from recently developed techniques such as identifying and studying quasiperiodic patterns (QPPs) of brain activity. QPPs, first introduced by the Keilholz group in 2009 (Majeed et al. 2009), refer to infraslow (0.01–0.2 Hz) spatiotemporal patterns in the BOLD signal that recur quasiperiodically throughout the duration of a resting-state scan. Interestingly, across multiple species, QPPs display prominent anticorrelation between the default mode network (DMN) and task-positive network (TPN) (Majeed et al. 2011; Abbas et al. 2016; Belloy et al. 2018b; Yousefi et al. 2018). The DMN and TPN are thought to regulate competing cognitive processes related to processing of internal and external input (Greicius et al. 2003; Fransson 2006; Northoff et al. 2010). Fluctuations in their activity reflect modulations in attention, affect sensory responses, and can explain some behavioral variability (Weissman et al. 2006; Helps et al. 2009; Sadaghiani et al. 2009; Esterman et al. 2013; Abbas et al. 2019). Specifically, time-varying DMN–TPN anticorrelations have been correlated with arousal fluctuations and lapses in behavioral performance (Thompson et al. 2013; Lynn et al. 2015; Wang et al. 2016). Substantial evidence thus suggests that QPP dynamics reflect fluctuations in brain state and may modulate task-evoked sensory responses, yet this question has not been formally investigated.

In this study, we hypothesized that the quasiperiodic anticorrelations between the mouse DMN- and TPN-like networks, identified under the form of QPPs (Belloy et al. 2018b), may reflect ongoing brain state fluctuations. In order to test this hypothesis, we performed fMRI experiments in healthy C57BL6/J mice under rest and sensory visual stimulation conditions and sought to answer if QPPs prior to a visual stimulus could explain variance in visual-evoked responses.

## Material and Methods

### Ethical Statement

All procedures were performed in strict accordance with the European Directive 2010/63/EU on the protection of animals used for scientific purposes. The protocols were approved by the Committee on Animal Care and Use at the University of Antwerp, Belgium (permit number 2017-38), and all efforts were made to minimize animal suffering.

### Animals

MRI procedures were performed on 24 male C57BL/6J mice (Charles River) between 18 and 22 weeks old. Animal handling and anesthesia procedures were similar to an established optimal light anesthesia protocol for mouse rsfMRI (Grandjean et al. 2014; Belloy et al. 2018b). Physiological parameters (respiratory and cardiac rate) were monitored for stability throughout scan sessions. Animals were scanned twice, 2 weeks apart (Supplementary Table S1). Additional details on animal procedures are provided in Supplementary Methods.

### MRI Procedures and Spatial Normalization

MRI scans were acquired on a 9.4T Biospec system (Bruker), with a 4-element receive-only phase array coil and volume resonator for transmission. For fMRI, whole-brain scans were acquired using gradient-echo echo-planar imaging (EPI) with a repetition time of 0.5 s. In each scan session, the first fMRI scan lasted 10 min and the directly consecutive fMRI scan (resting state or visual stimulation) lasted 15 min (Supplementary Fig. S1). Additionally, in each session, a 3D anatomical scan was also acquired. Study-based EPI and 3D anatomical templates were constructed, and the EPI template was normalized, in a 2-stage procedure (via 3D), to the Allen Brain Mouse Atlas (Oh et al. 2014). Further presented analyses of functional EPI data were thereby kept within the EPI template space. Additional details are provided in Supplementary Methods and Supplementary Figure S2.

### Visual Stimulation Design

Binocular visual stimulation with flickering light (4 Hz, 20% duty cycle) was presented to the animals by means of a fiber-optic coupled to a white LED, power-controlled by a digital voltage-gated device (Max-Planck Institute for Biological Cybernetics, Tübingen, Germany) and a RZ2 Bioamp Processor (Tucker-Davis technologies). Stimulation paradigms were triggered by a TTL pulse output from the scanner at the beginning of the EPI sequence. Visual stimulation fMRI scans lasted 15 min and visual stimuli were presented in a block design: 30 s ON, 60 s OFF, repeated 10 times with the first stimulus starting 30 s post scan start.

### Functional Scan Preprocessing

Preprocessing was as described previously and was implemented through Statistical Parametric Mapping software (SPM12; MATLAB2017b; Wellcome Department of Cognitive Neurology, London, UK) (Belloy et al. 2018b). A schematic overview is presented in Supplementary Figure S2. For visual-evoked fMRI scans, demeaning and variance normalization were performed with regard to 10-s OFF period prior to stimulation. All image-derived time series were therefore visualized in units of standard deviations (SD) from a zero-mean reference. Depending on the desired analysis, global signal regression (GSR) was performed. To determine spatiotemporal patterns, a brain mask was used to exclude ventricles.

### Spatiotemporal Pattern Finding Algorithm

QPPs were determined using the spatiotemporal pattern finding algorithm described by Majeed et al. (2011). Briefly, the algorithm identifies BOLD spatiotemporal patterns (distribution and propagation of BOLD activity across different brain areas over the duration of a specific predefined time window) that recur frequently over the duration of the functional scans. The process is unsupervised and starts by randomly selecting a starting template from consecutive frames in the image series, corresponding to the predefined time-window length. Then, this template is compared with the image series via sliding template correlation. A heuristic correlation threshold (ρ > 0.1 for the first 3 iterations and ρ > 0.2 for the rest) is used to define sets of images at peak threshold crossings that are averaged into a new template. This process is repeated until convergence. As the outcome of this procedure depends on the initial, randomly selected starting pattern, the process was repeated multiple times (*n* = 250) with randomly selected seed patterns from different time points in the time series. The process was also repeated for multiple window lengths (3–12 s, 1.5 s intersperse) as QPP length is not known a priori. QPPs were obtained by applying the algorithm to the concatenated time series of all individual subjects within a group. Detailed descriptions of the algorithm and videographic illustrations are provided elsewhere (Majeed et al. 2011; Belloy et al. 2018b).

### QPP Selection

After the spatiotemporal pattern finding algorithm concluded identifying the large set (*n* = 250 × 7 window sizes) of possible patterns, we proceeded to identify the patterns of interest based on prior knowledge, their similarity, and their correlation time series that indicate occurrences (correlation peaks) and time-varying similarity to the functional scans. It was previously established that both short (3 s) and long (9 s) QPPs can be uniquely identified from mouse (Belloy et al. 2018a, 2018b) and rat (Majeed et al. 2011) rsfMRI recordings. In these studies, short 3-s QPPs displayed the strongest time-varying correlation and were always marked by spatial anticorrelation of various brain areas, while longer QPPs displayed lower amplitude time-varying correlation, could also display brain-wide activity, and tended to capture biphasic extensions of shorter QPPs that have a lower probability of occurrence. Given these known priors, we opted to first identify 3-s QPPs. Then, QPPs were also defined for other window sizes. Specifically, for each window size, we selected as the most representative QPP the one that displayed the highest sum of correlation values at QPP occurrences (cf., Yousefi et al. 2018). From the resultant set of QPPs, the window size corresponding to a full cycle biphasic pattern was calculated (cfr., Belloy et al. 2018b). The window size for full-cycle QPPs was 9 s, consistent with the prior studies. All analyses were performed with and without GSR; findings for both approaches were integrated (cfr., below). Additional details are provided in Supplementary Methods.

### Phase–Phase Coupling

Contrary to conventional correlation-based approaches, phase–phase coupling analysis can be used to calculate whether signals display in-phase, out-of-phase, or antiphase properties. Prior work established that phase estimation for QPPs from rat rsfMRI data (Thompson et al. 2014), as well as for global signal and network fluctuations from mouse rsfMRI (Gutierrez-barragan et al. 2019), is feasible. Thus, for each subject respectively, the instantaneous phase of QPP or global signal time series were extracted using the Hilbert transform. Phase data were then binned across the [−π, π] range, and the number of matching observations between 2 respective signals was counted on phase–phase grids (normalized to scan length). Subject-specific effect estimates for each voxel on the grid were obtained using permutation statistics, shifting one of two time courses forward or backward in time [−10 s:0.5 s:10 s]. Group level significance maps were then obtained using one-sample *t*-tests, evaluated for each voxel respectively.

### QPP Significance Maps

The number of QPP occurrences (ρ > 0.2 threshold crossings) decreases with longer window sizes. Further, QPPs were determined with and without GSR. Therefore, to aid QPP comparisons, a homogenization procedure was employed. QPPs determined after GSR were correlated with image series for which no GSR was performed. The resultant correlation vector was used to calculate QPP occurrences. Further, after QPPs were defined, the correlation threshold (ρ > 0.2) was reduced for longer QPPs so that an equal number of occurrences were achieved as for short 3-s QPPs. For each QPP, *T*-scores were calculated for each voxel’s signal distribution of unique image frames contained within the QPP (*T* = $\mu /\Big(\sigma /\sqrt{n}\Big)$; μ = mean; σ = SD, *n* = number of image frames). Permutation statistics, randomly selecting an equal *n* of image frames, were used to obtain significance maps.

### Visual Activation Significance Maps

For each visual fMRI scan, the stimulation paradigm was convolved with a hemodynamic response function (HRF). The resultant visual predictor was used within a generalized linear model (GLM), that is, first-level analysis, to derive subject voxel-wise parameter coefficients (β) and *T*-values. Subject activation T-maps were then evaluated at the group level, that is, second-level analysis, by means of one-sample *t*-tests. The HRF was based on a literature-driven ground truth estimate (details in Supplementary Methods). Further, time frame by time frame group-average visual (de-)activation maps were also evaluated using one-sample *t*-tests at each voxel.

### Visual Response Analyses

The signal from visually activated areas (binary mask of significant group-level activations from GLM-based analysis), the global signal across all brain areas, and QPP correlation vectors were calculated for all subjects across all trials. Signal distributions at each respective time point of the trials were analyzed and visualized as peri-event time traces, mean- and variance normalized to the 10-s OFF period prior to stimulation. Activations (or deactivations) at each time point across trials [*n* = $90s/\Big(0.5\ \mathrm{s}/\mathrm{TR}\Big)$] were evaluated by one-sample *t*-tests.

### Visual Predictor Regression Analyses

For analyses in which the visual-evoked component was removed from the images, this was achieved by regressing the visual predictor signal (the convolution of the stimulation paradigm and the HRF) from each respective voxel and performing further analyses on the residual images.

### Phase-Based Visual Response Stratification

In order to determine whether QPPs could stratify differences in subsequent visual response magnitude, we utilized an analytical approach that uses only information regarding the phase of QPPs prior to stimulation and that is unbiased by stimulation paradigm or the visual responses.

Specifically, we estimated the instantaneous phase from QPP time series (using the Hilbert transform) on the 30-s time period just prior to each individual stimulation trial (when post-stimulus activity has essentially faded). Notably, because QPPs have a given temporal length (e.g., 3 s), the QPP correlation vector, at each of its time points, provides the correlation between the QPP and an equally long section of the fMRI image series. Therefore, to avoid bias from correlation of QPPs with the fMRI image series at trial transitions (e.g., correlation of a 3-s QPP with the fMRI image series 1 s prior to stimulation would incorporate a 2-s section of image data exposed to visual stimulation), correlations were only calculated between QPP and image sections prior to the transition time. Finally, in this process, correlation estimates become noisier closer to transition times, because fewer image time points are used for the correlation. To obtain the most robust phase estimate for the 3-s QPP just prior to stimulation, we used its phase 3 s prior to stimulation. For comparative purposes, we used the same 3 s prior timing for the 9-s QPP.

The phase of QPPs, just prior to stimulation, was then used to stratify stimulation trials into 3 groups, respectively, depending on whether the phase was high (HP; [$\pi /4$,$3\pi /4$]; i.e., “peaks”), low (LP; [−3$\pi /4$,−$\pi /4$]; i.e., “valleys”), or medium (MP; [3$\pi /4$,−$3\pi /4$] & [−$\pi /4$,$\pi /4$]; i.e., “slopes”). We reasoned that this reflects a physiologically meaningful stratification, that is, brain state dynamics may be affected differently by preceding state peaks, trough, or intermediary states (slopes). Groups were compared using one-way analysis of variance (ANOVA) tests for each respective time point during the stimulation period [*n* = $30\ \mathrm{s}/\Big(0.5\ \mathrm{s}/\mathrm{TR}\Big)$].

### Correlation of QPP and Reticular Formation Response Dynamics

To estimate covariance between QPPs and activity in the reticular formation (RF) during visual stimulation, Pearson correlations were calculated between their time series during the 5-s period after each stimulus onset, for all trials and animals, respectively. More specifically, because the RF displayed a faster transient peak than did QPPs, for each trial, the 5-s post-onset time series of the RF was first cross-correlated with the 10-s post-onset time series of the QPP. The lag corresponding to highest absolute cross-correlation value was then used to align the 5-s RF time series with the best matching 5-s QPP time series for subsequent Pearson correlation. By using absolute cross-correlation to guide the temporal alignment step, there is no bias for the directionality of correlation. The significance of QPP and RF covariance across all trials and animals was then determined using one-sample *t*-tests.

### Statistics

All permutation tests employed 1000-fold permutation in order to construct H0 distributions whereby *Z*-tests were subsequently used to evaluate true observations with regard to H0. All group-level significance maps were false discovery rate (FDR) corrected and cluster-size corrected (threshold = 4 voxels). All time-course analyses were FDR-corrected for the number of evaluated time points. ANOVA test was further Bonferroni corrected. For consistency, where applicable, *T*-values were standardized to *Z*-scores using the normal cumulative distribution function.

## Results

Experiments were performed in mice (*N* = 24) that were further separated in 2 equally populated groups (*N* = 12) that followed equivalent experimental procedures, resting state and visual fMRI, albeit with slightly different order to control for potential time and anesthesia effects (Supplementary Table S1). Overall, scan mean frame-wise displacement was negligible across all scans [0.038 ± 0.004 mm (mean ± STD)] (Supplementary Table S2). Resting-state scans from multiple sessions and time points were used to determine large-scale intrinsic connectivity networks (ICNs), by means of independent component analysis. These ICNs displayed clear physiological networks consistent with mouse literature (Liska et al. 2015; Zerbi et al. 2015), supporting data quality (Supplementary Fig. S3). There were no significant differences of ICNs between animal groups (Supplementary Table S3) or significant differences of visual activation maps between groups, supporting data pooling for subsequent analyses. An overview of the study design and all analyses is provided in Supplementary Figure S1.

### Quasiperiodicity during Resting State

**Figure 1:**
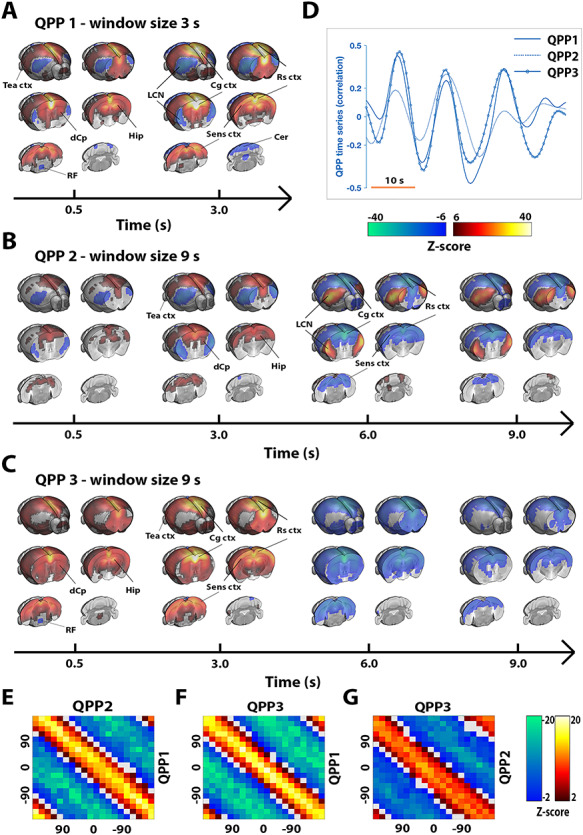
Three temporally colinear quasiperiodic brain fluctuations during resting state. Three QPPs were identified (*A*–*C*). QPP1 displayed a transient 3-s pattern capturing activation in DMN-like/sensory networks and deactivations in the LCN, QPP2 appeared similar as QPP1 but reverses in later frames, and QPP3 displayed cycling widespread activation and deactivation. Relevant brain areas are marked; DMN-like areas included Cg ctx, Rs ctx, Temporal association cortex (Tea ctx), Hip, and dCp. The 3 QPPs displayed a high degree of colinearity, evident both visually (*D*) and from phase–phase coupling (*E*–*G*). (*A*–*G*) *n* = 71 scans in 24 mice. (*A*–*C*) QPPs are displayed on the same time axis [alignment through cross-correlation of QPP correlation vectors (*D*)]. Maps display *Z*-scores [*Z*-test with H0 through randomized image averaging (*n* = 1000), FDR *P* < 10^−7^]. (*D*) Single subject excerpt. QPP correlation vectors represent Pearson correlations of QPPs with functional image series. (*E*–*G*) Phase–phase plots show *Z*-scores and are constructed from QPP correlation time series; center hot (yellow and red) diagonal marks strong co-phasic dynamics [first level *Z*-test with H0 through randomized circular shuffling (*n* = 1000); second level *Z*-test, FDR *P* < 0.05]. Hip, hippocampus; dCp, dorsal caudate putamen; Cg ctx, cingulate cortex; Rs ctx, retrosplenial cortex; Sens ctx, sensory cortex; Cer, cerebellum.

Using previously established analysis strategies (cfr., Belloy et al. 2018a, 2018b; M&M), we consistently identified 3 QPPs of interest in the data (Fig. 1*A*–*C*): QPP1, a short 3-s pattern that displayed a transient widespread anticorrelation between DMN-like/sensory networks and the lateral cortical network (LCN; a proposed mouse analogue of the TPN; cfr., Discussion) (Supplementary Video 1); QPP2, a 9-s pattern that initially is similar to QPP1 but continues and reverses pattern in later frames (Supplementary Video 2); and QPP3, a 9-s pattern cycling between widespread activation and deactivation (Supplementary Video 3). Except for the LCN, the 3 QPPs largely involved the same brain areas. All 3 QPPs displayed a high degree of temporal colinearity (Fig. 1*A*–*D*), suggesting that they may have captured different components of a shared process, which could not be uniquely identified as a single spatiotemporal pattern. To further estimate the time relationship of these 3 QPPs, phase–phase plots were constructed for all QPP pairs (Fig. 1*E*–*G*). All QPPs displayed prominent phase–phase coupling, and this was only somewhat reduced between QPP2 and QPP3. Notably, for each of the observed QPPs, an opposite phase variant was also observed with consistent temporal characteristics (Supplementary Fig. S4). These were not further considered, given their equivalence (nearly inverted time series) to the primary described QPPs.

### Intrinsic Quasiperiodic Brain Dynamics Stratify Visual Response Magnitude

**Figure 2:**
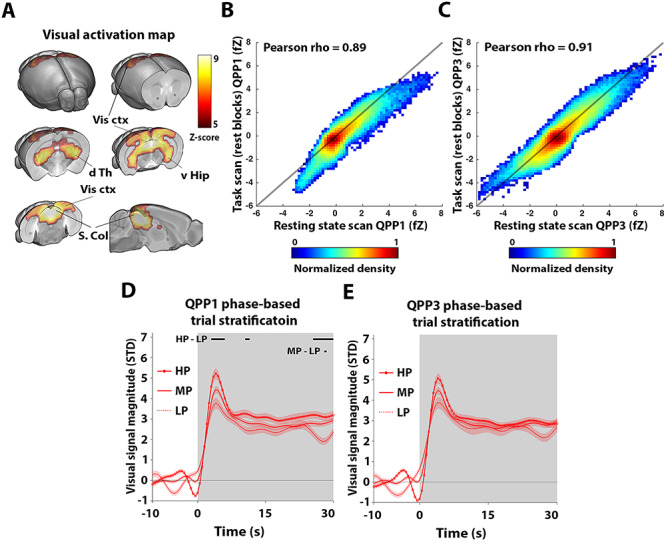
Phase of quasiperiodic brain fluctuations prior to stimulation stratify subsequent visual response magnitudes. Reliable visual activations were observed in brain areas related to visual sensory processing (*A*). A short 3-s QPP, highly similar to QPP1 determined during resting state scans, was observed during the rest blocks prior to stimulation in task scans (*B*). A consistent 9-s QPP was also observed (*C*). The phase of QPP1 (which displays activations in DMN-like areas) just prior to stimulation was used to stratify trials into 3 groups, respectively, depending on whether the phase was high (HP; [$\pi /4$,$3\pi /4$]; i.e., “peaks”), low (LP; [−3$\pi /4$,−$\pi /4$]; i.e., “valleys”), or medium (MP; [3$\pi /4$,−$3\pi /4$] & [−$\pi /4$,$\pi /4$]; i.e., “slopes”). This highlighted significant stratification of visual signal amplitudes by QPP1 at the transient response peak and end of the stimulation block (black bars in *D*). Using the phase of QPP3 prior to stimulation achieved similar stratifications as obtained for QPP1, but these were not significant (*E*). (*A*–*E*) *n* = 24 scans in 24 mice. (*A*) Maps display *Z*-scores (first level GLM; second level one-sample *t*-test; *T*-scores normalized to *Z*-scores; FDR *P* < 10–5]. (*B*, *C*) Scatter density plots of QPP fZ image intensities. (*D*, *E*) Gray areas mark trials (ON periods), traces show mean, and patches show standard error. Time traces are demeaned and variance normalized to 10-s OFF period prior to stimulation. Black bars indicate significant differences between trial groups [one-way ANOVA, FDR (#bins) *P* < 0.05; post hoc Bonferroni correction]. v Hip, ventral hippocampus; d Th, dorsal thalamus; Vis ctx, visual cortex; S. Col, superior colliculus; fZ, fisher *Z*-transformed.

After determining QPPs in resting-state conditions, we sought to establish if similar QPPs can also be observed during a visual stimulus processing design that is expected to trigger changes in brain state. To this end, we used a visual stimulation block design (30 s ON−60 s OFF) with intentionally long OFF periods to allow the activity to return to baseline each time before the next visual activation block. First, to identify the visually stimulated areas, we used a classical GLM approach by convolving the block-design paradigm with the HRF in order to derive the signal response predictor (cfr., M&M). Clear activations were observed in areas related to visual processing: dorsal thalamic nuclei (including the lateral geniculate nucleus), superior colliculus, visual cortex, and hippocampus (Fig. 2*A*). These activation maps were highly consistent with those previously reported in mice, supporting data quality (Niranjan et al. 2016).

Then, the QPP spatiotemporal pattern finding algorithm was used to determine if spatiotemporal patterns similar to resting-state QPPs could be observed in the visual fMRI scans. In this case, in addition to the normal analysis, we also performed the QPP estimation after performing GSR, which we reasoned could potentially remove brain-wide responses induced by visual stimulation that would interfere with QPP detection. Both with and without GSR, the resultant spatiotemporal patterns were largely dominated by visual activations, and also brain-wide responses in prefrontal and lateral cortical areas, but they were not clearly reminiscent of resting-state QPPs (Supplementary Fig. S5).

To eliminate spatiotemporal patterns that directly reflect visual activation, we also performed the same analysis under 2 other conditions: 1) after the visual predictor was regressed from the task fMRI scans and 2) applying the pattern finding algorithm solely to all 30-s rest periods preceding stimulation blocks. Under both these conditions, the spatiotemporal pattern finding algorithm revealed a 3-s QPP that was highly similar to QPP1 in resting-state scans (spatial cross-correlation = 0.89; Fig. 2*B*) and a 9-s QPP highly similar to QPP3 in resting-state scans (spatial cross-correlation = 0.91; Fig. 2*C*). No pattern similar to QPP2 was observed (Supplementary Fig. S5). Given these findings, we reasoned that QPP1 and QPP3 were present in the (rest periods of) task fMRI scans and we further studied their signal properties.

To address our primary research question—whether QPPs prior to a visual stimulus could explain variance in visual-evoked responses—we investigated if the phase of QPP time series just prior to stimulation could stratify stimulation trials into varying levels of response magnitude. Specifically, trials were grouped into high (HP), low (LP), and medium phase (MP) groups (cfr., M&M). Interestingly, the phase of QPP1 could significantly stratify subsequent visual response magnitude differences at the peak of the visual response and toward the end of the 30-s stimulation blocks (Fig. 2*D*). The HP trials group was marked by an overall higher response magnitude compared with the LP group. Despite that QPP3 displayed a similar phase-based stratification of visual responses, it could not stratify significant differences between trial groups (Fig. 2*E*). When, for QPP1, these analyses were broken down per brain area (as indicated in Fig. 2*A*), all areas displayed similar effects as observed for the full visual activation map, but significant stratification was only observed for visual cortex and hippocampus at the peak of the visual response (Supplementary Fig. S6).

To support these findings, we also confirmed that there were no sensory habituation effects or variability in response distributions across animals and trials. Visual response magnitude in preceding trials had no bearing on visual response magnitude in subsequent trials (Supplementary Fig. S7*A*) nor did QPP phase allow for significant stratification of visual response magnitude in preceding trials (Supplementary Fig. S7*B*). Visual responses across subsequent trials appeared consistent, and visual response distributions across trials and animals were largely similar (Supplementary Fig. S8).

### Quasiperiodic Brain Patterns Display Response Properties during Stimulation

**Figure 3:**
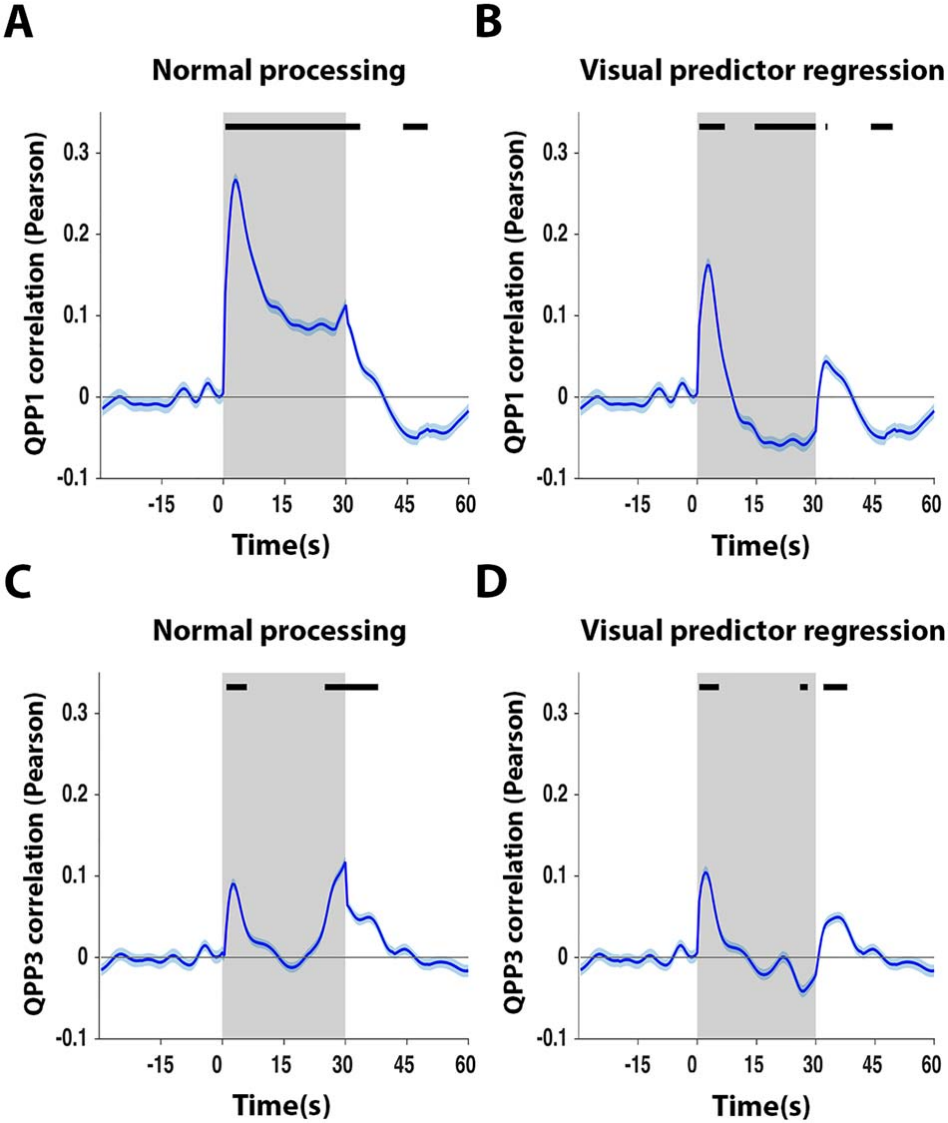
Quasiperiodic brain fluctuations display intrinsic response dynamics to stimulation. QPP1 and QPP3 displayed, on average, a peak correlation at the start of stimulation trials (*A*,*B*), which persisted even after regression of the visual predictor (*C*,*D*), potentially suggesting that QPPs displayed an intrinsic response component rather than visual processing per se. (*A*–*D*) *n* = 24 scans in 24 mice. Gray areas mark trials (ON periods), traces show mean, and patches show standard error. Time traces are demeaned and variance normalized to 10-s OFF period prior to stimulation. QPP correlation vectors (blue) are respectively averaged across all trials and animals (*n* = 10 trials × 24 animals). Black bars mark significance (one sample *t*-test, FDR *P* < 10^−5^).

Surprisingly, when visualizing trial-locked QPP time series in the task scans, QPP1 and QPP3 displayed, on average, a significantly increased correlation with the image series, particularly (and especially for QPP1) at the start of visual stimulation blocks (Fig. 3*A*,*B*). Notably, the increased correlation of the QPPs around the start of the visual stimulation was preserved after regression of the visual stimulation predictor (Fig. 3*C*,*D*). We therefore conjectured that the QPPs may have represented an intrinsic component triggered by the visual stimulus but did not represent the visual sensory processing per se (cfr., Discussion for limitations). This was further supported by the higher spatial correlation of task scan QPP1 with the resting-state scan QPP1 (spatial cross-correlation = 0.89; Fig. 2*B*) in comparison with the visual activation profile (spatial cross-correlation = 0.56 when excluding significantly activated areas; cfr., Fig. 2*A*) and, in addition, by the fact that the QPPs were also observed at different time points beyond the start of the visual stimulation blocks, such as during off periods and occasionally at different times during the visual stimulus (Fig. 2*B* and Supplementary Fig. S9).

### Colinearity with Fluctuations in the RF

**Figure 4:**
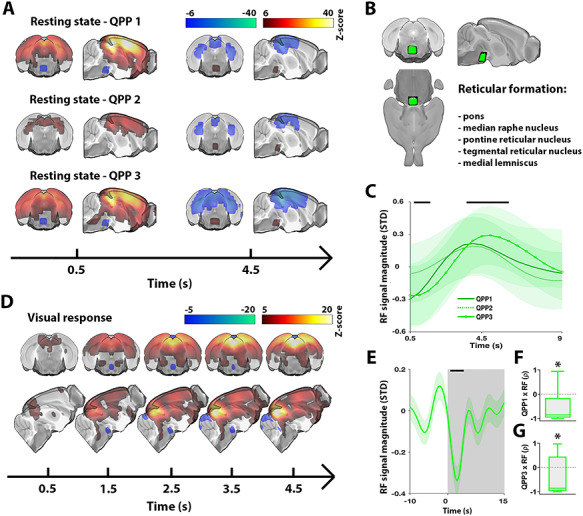
Activity in the RF covaries with quasiperiodic brain dynamics across the rest/task spectrum. All QPPs displayed significant activity in a focal dorsal brain stem area (*A*). Anatomical labeling through coregistration with the Allen Mouse Brain Atlas highlighted that this area contained nuclei of the RF (*B*). Time courses of the RF were on average highly similar across investigated spatiotemporal patterns (*C*). The RF also displayed deactivation at the start of stimulation blocks (*D*, *E*). Furthermore, the RF and QPP responses at the start of stimulation were significantly correlated, more so for QPP1 than QPP3 (*F*, *G*). (*A*–*C*) *n* = 71 scans in 24 mice. (*A*) Maps display *Z*-scores [*Z*-test with H0 through randomized image averaging (*n*=1000), FDR *P* < 10^−7^]. Time points were chosen to visualize when signal dynamics in the RF passed the significance threshold (as in *C*). The full temporal extent of significant RF dynamics can be appreciated in Supplementary Videos 1–3. (*B*) Visual rendering of focal brain area observed in (*A*) and (*D*). List indicates anatomical structures contained within this area. (*C*) Average RF time series across respective QPP correlation peaks (traces show mean; patches show standard error). Black bars mark significant deviation from zero (statistical test as in *A*) observed in at least one QPP. (*D*) Visual response averaged across trials and animals (*n* = 10 trials × 24 animals) for first 5 s of stimulation. Voxel-wise time courses were demeaned and variance normalized to 10-s OFF period prior to stimulation. Maps display *Z*-scores (one sample *t*-test; *T*-scores normalized to *Z*-scores; FDR *P* < 10^−5^). (*E*) Gray areas mark trials (ON periods), trace shows RF area signal mean, patch shows standard error, and black bar marks significance (one sample *t*-test, FDR *P* < 10^−5^). (*F*, *G*) Box plots of Pearson correlations between RF and QPP signals at the start of each visual stimulation trial (*n* = 10 trials × 24 animals) (one sample *t*-test, ^*^P < 0.05). ρ, Pearson correlation*.*

Previously, it has been suggested that the brain dynamics observed in QPPs may be orchestrated through neuromodulation (Majeed et al. 2011). Similarly, in the lieu of global brain dynamics—to which QPPs relate (Keilholz 2014; Belloy et al. 2018b; Yousefi et al. 2018)—there have been multiple studies showing that neuromodulatory nuclei are focally deactivated and play a regulatory role during brain-wide activations (Liu et al. 2018; Turchi et al. 2018). Interestingly, detailed observation of the QPPs in our study serendipitously unveiled that a focal area at the dorsal part of the brain stem cycled antagonistically with overall brain-wide activity (Fig. 4*A*). To identify the cytoarchitectonic location of this area, we coregistered the MRI data to the Allen mouse brain atlas. This revealed that this area contained mainly pontine nuclei of the RF (Fig. 4*B*). The average RF time courses across all 3 QPPs were highly similar (Fig. 4*C*), with an initial significant dip, followed by a significant peak approximately 4.5 s later.

To understand if activity in the RF could be related to the apparent stimulus-evoked observation of QPPs during visual stimulation, we plotted the average initial time frames of the event-related activation maps (Fig. 4*D*). Interestingly, significant deactivations in the RF were observed time-locked to the start of visual stimulation. The time course of RF activity is presented in Fig. 4*E*. Furthermore, the response dynamics of the RF and QPPs were also significantly correlated, supporting that the RF may play a regulatory role in QPPs (Fig. 4*F*,*G*).

## Discussion

In this study, we showed that QPPs, which are marked by large brain-wide activations and anticorrelation between the DMN- and TPN-like networks, are the predominant spatiotemporal patterns observed during resting conditions in lightly anesthetized mice. We then showed that the pre-stimulus phase of a short 3-s QPPs, which displayed activations of the DMN-like network and deactivations of the TPN-like network, captured variance in subsequent visual response magnitude. QPPs also displayed stimulus response properties not accounted for by a visual signal response predictor, potentially indicating an intrinsic brain response. Supporting this finding, we observed that QPPs displayed co-phasic activity in a focal area of the RF, a major regulator of neuromodulation, arousal, and brain state. Specifically, the transient onset response properties of QPPs during visual stimulation covaried with deactivations in the same focal area of the RF. In summary, our findings suggest that QPPs capture a brain state fluctuation that may be orchestrated through neuromodulation and affects sensory processing.

QPPs observed here were highly consistent with those observed in previous mouse studies using single slice recordings (Belloy et al. 2018a, 2018b). Specifically, QPP1 and QPP2 displayed widespread anticorrelation between the commonly observed mouse LCN and DMN-like/sensory networks (Liska et al. 2015; Zerbi et al. 2015; Grandjean et al. 2017). So far, a mouse TPN-like network has not been clearly identified, but the LCN has been suggested as the most likely candidate (Liska et al. 2015; Zerbi et al. 2015). We therefore discuss the LCN interchangeably with “mouse TPN-like network” and observe that QPPs displayed anticorrelation of the DMN- and TPN-like networks. Further, all QPPs displayed a high degree of temporal colinearity, suggesting that they likely reflected variants in a single spatiotemporal pattern. This is consistent with prior observations (Majeed et al. 2011; Belloy et al. 2018b), whereby shorter QPPs occurred more frequently while longer QPPs identified instances where short QPPs oscillated and reversed in later frames. The network dynamics observed for QPP1 and QPP2 also display consistency with the quasioscillatory dynamics of coactivation patterns (CAPs, i.e., instantaneous brain activity patterns) that display DMN activation and TPN deactivation, as observed in humans (Liu and Duyn 2013), rats (Zhang et al. 2020), and mice (Gutierrez-barragan et al. 2019). Notably, the longer QPP2 was not observed in visual stimulation scans, suggesting that the overall brain state during our task design lowered the probability of observing oscillatory brain dynamics.

We observed that QPPs, displaying DMN activations and TPN deactivations, just prior to visual stimulation could be used to stratify subsequent response magnitude in visually evoked areas. Our study is thereby the first to show in mice that fluctuations of the DMN- and TPN-like networks, and particularly their anticorrelation, capture a brain state dynamic that affects sensory processing. Similar findings based on DMN and TPN fluctuations, or their anticorrelation dynamics, were previously reported in human studies (Sadaghiani et al. 2009; Thompson et al. 2013). Interestingly, it has been proposed that rhythmic anticorrelations of the DMN and TPN modulate the brain state between attentional lapses and periods of improved sensory entrainment, which in turn would explain differences in evoked responses in sensory brain areas (Lakatos et al. 2016). This could help understand how QPPs were able to stratify the subsequent visual response magnitude in our study. It currently still remains unclear into what extent DMN- and TPN-like dynamics during a task in mice would be comparable to those in humans or nonhuman primates. Our findings indicate there appears to be at least some preserved functional homology between these species, suggesting that future mouse fMRI studies will be valuable for the study of brain state dynamics.

Surprisingly, QPPs, marked by deactivation of the TPN-like network and activation of the DMN-like/sensory networks, also displayed stimulus-related responses. This apparent task-related DMN activation may be considered counterintuitive with regard to conventional observations that task engagement causes decreased DMN activity and increased TPN activity (Fransson 2006; Northoff et al. 2010). In contrast, other studies reported a less canonical role of the DMN that is more consistent with the current findings (Sadaghiani et al. 2009; Esterman et al. 2013; Kucyi et al., 2016, 2017). In the latter, DMN activity during a task reflected an attentive state, while TPN activity was associated with increased behavioral variance and suppressed attention. Extrapolating on this, under anesthetized conditions in mice, it may be more likely that QPP responses to stimulation could reflect an intrinsic arousal-related component. Most mouse fMRI studies to date, which focused primarily on somatosensory stimulation, observed brain-wide responses that were at least partially due to increases in mean arterial blood pressure, caused by the noxious nature of presented stimuli (Schlegel et al. 2015; Schroeter et al. 2016; Reimann et al. 2018). However, in our study, we did not observe such responses under the employed visual stimulation and anesthesia protocols. The apparent QPP dynamics in response to visual stimulation are therefore unlikely to correspond to physiological parameters during the recordings. QPPs have furthermore been related to a neuronal substrate (Pan et al. 2013; Grooms et al. 2017), while no clear link between QPPs and cardiovascular or respiratory physiology could be established in prior mouse, rat, and human work (Majeed et al. 2011; Belloy et al. 2018b; Yousefi et al. 2018). An important limitation for the apparent stimulus-related QPP response is that it may in fact reflect residual visual activation that could not be modeled by the visual predictor. However, we specifically employed a more realistic mouse HRF, consistent with fast hemodynamics in the mouse brain (Drew et al. 2011; Pisauro et al. 2013). Furthermore, given the observation of covariation between “evoked” QPPs and deactivation in the RF (cfr., below), our findings suggest that QPP responses may truly reflect an intrinsic neuronal component rather than residual visual activations.

QPPs displayed colinear activity in a focal brain stem area comprising core nuclei of the RF. The ascending reticular activating system, which comprises the RF, is responsible for promoting wakefulness and attention through the orchestrated activity of neuromodulatory nuclei, such as raphe nucleus, locus coeruleus, and nucleus basalis. These neuromodulatory structures are also natural rhythm generators that provide infraslow patterned input to the brain (Drew et al. 2008) and have previously been proposed as potential orchestrators of QPPs (Keilholz 2014). In humans, these nuclei have been functionally connected to the DMN (dorsal raphe nucleus) and TPN (locus coeruleus) (Bär et al. 2016). In mice, optogenetic activation of the serotonergic dorsal raphe nucleus caused widespread deactivation of DMN-like areas (Grandjean et al. 2019), which reflected the QPPs observed in the current study. The observation of deactivation in the RF thus supports that QPPs may arise from the patterned input of neuromodulatory nuclei to the brain. Furthermore, neuromodulation allows adaptation of brain state to modulate processing of sensory stimuli (Lee and Dan 2012; Safaai et al. 2015). This could explain the transient response properties of QPPs and covarying deactivation in the RF during visual stimulation. Future experiments will be required to tease out the potential neuromodulatory regulation of QPPs using tools such as optogenetics (Carter et al. 2010).

Notably, QPP3 appeared as a global brain-wide activation and was also temporally colinear with QPP1 and QPP2. We have previously made similar observations in both mice and human (Belloy et al. 2018b; Yousefi et al. 2018), while a recent independent study in mice congruently showed that CAPs with DMN-like activations and TPN-like deactivations are mainly observed during the peak of global signal fluctuations (Gutierrez-barragan et al. 2019). Furthermore, the nucleus basalis was shown to be deactivated during global brain signal peaks in humans (Liu et al. 2018), which is reminiscent of the deactivations that we observed in the RF across all QPPs. Given these observations, we performed a series of supplementary analyses to determine shared properties between QPPs and the global signal. We observed that the global signal and QPPs displayed strong temporal colinearity (Supplementary Fig. S10). Further, we observed that the global signal displayed the same focal deactivation in the RF as QPPs did, that the global signal prior to visual stimulation could stratify subsequent visual response magnitude similar as was the case for QPP1, and that the global signal also displayed stimulus-related response properties (Supplementary Fig. S11). These observations support our main findings, as global signal fluctuations have been reported to reflect changes in arousal state (Liu et al. 2017), to be regulated by neuromodulation (Schölvinck et al. 2010; Turchi et al. 2018), and to modulate sensory responses (Lee and Dan 2012; Mcginley et al. 2015; Schölvinck et al. 2015; Pisauro et al. 2016). Additionally, these findings also illustrate how QPPs allowed novel insights into network and brain state properties that are not obvious from global signal dynamics alone. Specifically, considering the observed phasic relationship between QPPs and the global signal, QPPs could actually capture spatial topography contained within global brain fluctuations. This is important because it informs us that network dynamics (i.e., DMN/TPN-like) in QPPs and global brain activity may share similar underlying mechanisms, which is supported by our observation of a similar temporal relationship with the RF. Tracking QPPs together with the global brain signal may thus allow future studies to further disentangle the mechanisms that regulate brain state dynamics. Furthermore, the global signal in most studies is composed of several components, including noise, physiological fluctuations, neuronal activity related to arousal, and true resting-state network dynamics (Liu et al. 2017; Turchi et al. 2018). In this study, the global signal showed colinear dynamics with QPPs supporting that in cases where no significant motion is present and physiology is stable, it also contains substantial information reflecting neural activity related to arousal and resting-state networks.

In summary, we show for the first time in mice that DMN- and TPN-like network fluctuations, and particularly their anticorrelation, capture a brain state dynamic that affects sensory processing, and that this brain state can be identified under the form of QPPs. Our findings support the hypothesis that quasiperiodic anticorrelations of the DMN and TPN reflect modulations in brain arousal state and suggest that the RF may play an important role in mediating this effect. Our study provides new frontiers to understand the neural processes that shape functional brain states and suggests that mouse fMRI studies represent a promising platform in this research field.

## Supplementary Material

Belloy2020_QPP_supplementary_09_16_cerebral_cortex_bhaa305

Video1_bhaa305

Video2_bhaa305

Video3_bhaa305

Video_captions_bhaa305

## Notes

The computational resources and services used in this work were provided by the HPC core facility CalcUA of the Universiteit Antwerpen, the VSC (Flemish Supercomputer Center) and funded by the Hercules Foundation and the Flemish Government—Department EWI. *Conflict of Interest*: The authors declare no competing interests.
