# Supplementary material for: Resting Brain Fluctuations Are Intrinsically Coupled to Visual Response Dynamics
Source: Cereb Cortex. 2020 Oct 27;31(3):1511–22. doi: 10.1093/cercor/bhaa305 (PMC7869084; doi:10.1093/cercor/bhaa305)
Supplement: Belloy2020_QPP_supplementary_09_16_cerebral_cortex_bhaa305 [file belloy2020_qpp_supplementary_09_16_cerebral_cortex_bhaa305.docx]

**Resting brain fluctuations are intrinsically coupled to visual response dynamics**

**Authors:**

Michaël E. Belloy^1-2^, Jacob Billings^3^, Anzar Abbas^3^, Amrit Kashyap^2^, Wen-ju Pan^2^, Rukun Hinz^1^, Verdi Vanreusel^1^, Johan Van Audekerke^1^, Annemie Van der Linden^1^, Shella D. Keilholz^4^, Marleen Verhoye^1^, Georgios A. Keliris^1^.

**Authors’ affiliations:**

**^1^** Bio-Imaging Lab, Department of Pharmaceutical, Veterinary and Biomedical Sciences, University of Antwerp, Universiteitsplein 1, 2610 Wilrijk, Antwerp, Belgium.

**^2^** Department of Biomedical Engineering, Emory University and Georgia Institute of Technology, 1760 Haygood Dr NE, Atlanta, GA, 30322, USA

**^3^** Department of Neuroscience, Emory University, 1760 Haygood Dr NE, Atlanta, GA, 30322, USA.

***Corresponding authors:**

M.E.B. [Michael.belloy@hotmail.be](mailto:Michael.belloy@hotmail.be)

S.D.K. [Shella.keilholz@bme.gatech.edu](mailto:Shella.keilholz@bme.gatech.edu)

G.A.K. [Georgios.keliris@uantwerpen.be](mailto:Georgios.keliris@uantwerpen.be)

**Supplementary Material**

**Supplementary Methods**

**Animal handling in the scanner**

Animals were anesthetized with 3.5% isoflurane and maintained at 2% during handling. Heads were fixed with bite and ear bars. Moldable earplugs were positioned on top of the ear bars in order to provide hearing protection. Ophthalmic ointment was applied to the eyes. Animal core body temperature was measured with a rectal temperature probe and kept stable at 37°C via hot air supply (MR-compatible Small Animal Heating System, SA Instruments, Inc.). A pressure sensitive pad was used to assess breathing rate, and a fiber-optic pulse oximeter placed over the tail, to assess heart rate and O_2_ saturation (MR-compatible Small Animal Monitoring and Gating system, SA Instruments, Inc.). Animals were anesthetized with a 0.075mg/kg bolus subcutaneous injection of medetomidine (Domitor, Pfizer, Germany), after which isoflurane was gradually lowered to 0.5%. A subcutaneous catheter allowed continuous infusion of 0.15mg/kg/h medetomidine starting 15min post-bolus. Acquisition of functional scans started 30min post-bolus.

**MRI procedures and spatial normalization**

Rapid Acquisition with Refocused echoes (RARE) images were acquired in three orthogonal directions (repetition time (TR) 2500ms, effective echo time (TE) 33ms, 16 slices of 0.4 mm), to enable reproducible slice positioning. B0 field maps were acquired, followed by local shimming. Functional MRI scans were acquired with a gradient-echo EPI sequence (field of view (27x21x6) mm^3^, matrix dimensions [90x70x12], slices thickness 0.4mm, slice intersperse 0.1mm, voxel-resolution (0.3 x 0.3 x 0.4) mm3, flip angle 55°, bandwidth 250kHz, TR 500ms, TE 14ms). In each scan session, a T2-weighted 3D anatomical scan was acquired (RARE, TR 1800ms, TE 6ms, RARE factor 16, spatial resolution (0.078 x 0.078 x 0.31) mm^3^).

The open source registration toolkit ANTs was used to construct a study-based 3D T2 RARE template and a 2D EPI-based study template. The 2D EPI-based study template was then registered to the Allen brain atlas^1^ with ANTs using a two-stage registration procedure. First, registration of the masked and debiased 3D study template to the atlas was performed, and second, registration of the masked and debiased 2D EPI study template to the 3D study T2 RARE template was performed. Using the 2-stage registration parameters, EPI template space and related significance maps were forward-transformed into atlas space.

**Frame-wise displacement**

Frame-wise displacement (FD) was calculated at each point by taking the sum of absolute backwards looking temporal derivatives for all three motion time series^2^. To compute rotational displacement and convert degrees to millimetres, an assumption was made where the mouse brain is considered as a sphere with a diameter of 10mm.

**Resting state independent component analysis**

To obtain intrinsic connectivity networks (ICN), group ICA was performed on all rsfMRI scans (n = 71; **Supplementary Table 1**) using the GIFT toolbox (v4.0b) (Calhoun et al., 2004). The number of independent components was set to N=5. If the number of components was increased beyond five, anti-correlation diminished and the DMN became split into an anterior and posterior component. The ICA was run on variance-normalized data (z-scoring procedure), filtered between 0.008-0.2Hz, using the Infomax algorithm with no auto-filling of data reduction values. Stability analysis was performed using the ICASSO algorithm, rerunning the ICA 20 times with a minimal cluster size of 10 and maximal of 20. Using the built-in GIFT functionality, SPM12 was used to obtain T-contrast images per subject for all components. Second level one sample T-tests were performed for all components to obtain significant group level ICNs [p<10^-5^, FDR, cluster-correction 4 voxels).

**Quasi-periodic pattern selection – determining window size**

For each set of QPPs, at a respective window size, QPPs were first grouped into opposing phase observations. Opposing phase implies that within a set, QPPs displayed similar involvement of spatial areas, but with inverted timing of activation and deactivation. This heuristic assumption was inspired by prior observations^3-4^, and overall visual inspection of the current findings. In order to phase QPPs, the signal of a specific set of brain areas over the duration of the QPP was used. Specifically, DMN-like areas have been consistently observed as a component of QPPs. Therefore, a mask of the DMN-like ICN was derived from the ICA analysis (**Figure S1**) and was used to average the intensity of all DMN voxels at each time frame within the QPPs. Using these time courses, QPPs were phase sorted according to a previously described strategy [cfr. (Belloy et al., 2018)^3^]. From the phase sorted groups at each window size, a single QPP was obtained by selecting the one that displayed the highest value of summed QPP correlation vector peaks [cfr. (Yousefi et al., 2018)^5^]. As QPPs at smaller window sizes are expected to be parts of longer bi-phasic QPPs, a previously described algorithm was used to select the most representative window length for the identified QPPs^3^. This algorithm identifies the amount of overlap between QPPs of different lengths, termed the fractional average correlation (FA), by calculating image correlation at different temporal lags. Then, it identifies the window length after which longer variants of the same QPP no longer substantially increase the FA value. This determines the most representative window length and allows selection of QPPs for further investigation. For the current study, this cut-off point was 9s.

**Haemodynamic response function**

Currently, no true mouse HRF is available in the literature, but prior work has indicated fast and sharp neurovascular coupling in the mouse brain^6-8^. Particularly, the observations made by Lebhardt and colleagues (2015) were here parameterized by a mixture of two gamma variate functions within SPM, using the function “spm_hrf” (settings: response delay = 2s, undershoot delay = 6s, response dispersion = 0.5, undershoot dispersion = 1, response/undershoot ratio = 12, onset = 0s, kernel length = 6s).

**Global signal co-activation pattern**

To visualize the global signal (**Figure S5**), image frames surrounding global signal peaks were averaged into a spatiotemporal template, i.e. a global signal co-activation pattern (CAP). This approach is consistent with the methodology presented by Liu and Duyn (Liu and Duyn, 2013)^9^, but includes temporal extension of signal peaks. A detailed description of this method is described elsewhere (Belloy et al., 2018)^3^. An activation map of the global CAP, and related statistical analysis, was calculated in the same way as described for QPPs (cfr. main text).

**Elongation of quasi-periodic patterns**

Image frames that followed/preceded the core of short QPPs (e.g. 3s) were included to allow comparison with other longer spatiotemporal patterns (**Figure S2**). In this procedure, there is no re-estimation of the QPP or its correlation vector, only additional image frames following correlation peaks are averaged into the elongated template.

**Supplementary references**

1. Oh SW, et al. (2014) A mesoscale connectome of the mouse brain. *Nature* 0(1). doi:10.1038/nature13186.

2. Power JD, Barnes KA, Snyder AZ, Schlaggar BL, Petersena SE (2012) Spurious but systematic correlations in functional connectivity MRI networks arise from subject motion. *Neuroimage* 59(3):2142–2154.

3. Belloy ME, et al. (2018) Dynamic resting state fMRI analysis in mice reveals a set of Quasi-Periodic Patterns and illustrates their relationship with the global signal. *Neuroimage* 180, Part:463–484.

4. Belloy ME, et al. (2018) Quasi-Periodic Patterns of Neural Activity improve Classification of Alzheimer’s Disease in Mice. *Sci Rep* 8(1). doi:10.1038/s41598-018-28237-9.

5. Yousefi B, Shin J, Schumacher EH, Keilholz SD (2018) Quasi-periodic patterns of intrinsic brain activity in individuals and their relationship to global signal. *Neuroimage* 167:297–308.

6. Kahn I, et al. (2011) Characterization of the Functional MRI Response Temporal Linearity via Optical Control of Neocortical Pyramidal Neurons. *J Neurosci* 31(42):15086–15091.

7. Lebhardt P, Von Hohenberg CC, Weber-fahr W, Kelsch W, Sartorius A (2015) Optogenetic fMRI in the mouse hippocampus : hemodynamic response to brief glutamatergic stimuli. *J Cereb Blood Flow Metab* 36(3):629–638.

8. Drew PJ, Shih AY, Kleinfeld D (2011) Fluctuating and sensory-induced vasodynamics in rodent cortex extend arteriole capacity. *Proc Natl Acad Sci U S A* 108(20):8473–8478.

9. Liu X, and Duyn JH (2013) Time-varying functional network information extracted from brief instances of spontaneous brain activity. Proc. Natl. Acad. Sci. *110*, 4392–4397.

**Supplementary tables and figures**

**Table S1. Overview of experimental design.** Matching colours mark consistent scan types.

**Table S2. Mean frame-wise displacement.** Colour codes in Table S1. For one animal, a scan reconstruction error occurred (red text).

**Table S3. Group comparison.** Each compartment indicates tests that compared similar scan types. These were evaluated to determine if data pooling was appropriate. No images are shown, because no significant differences were apparent on networks or activation maps.

**
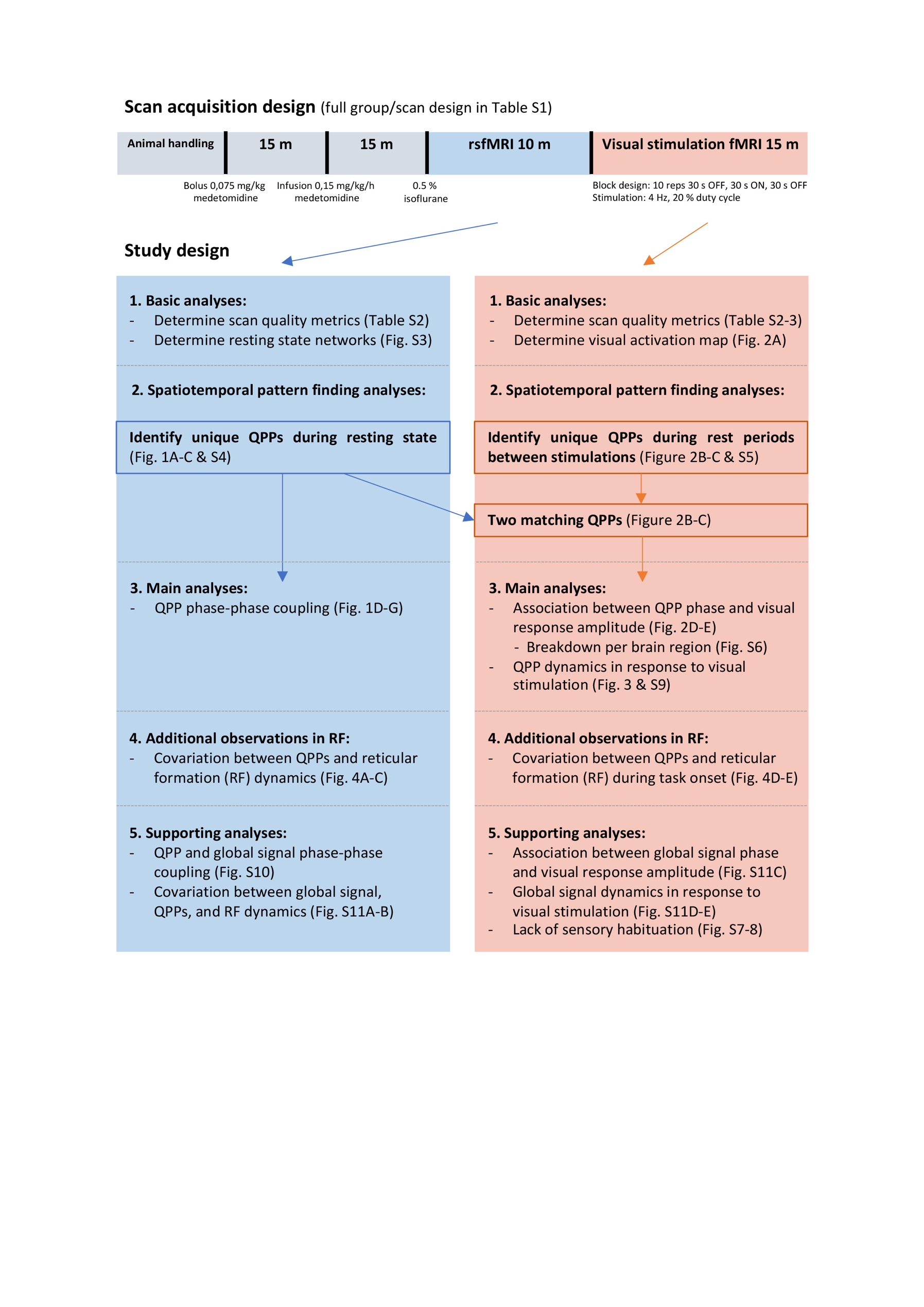
**

**Figure S1. Overview of scan acquisition and study design.**

**
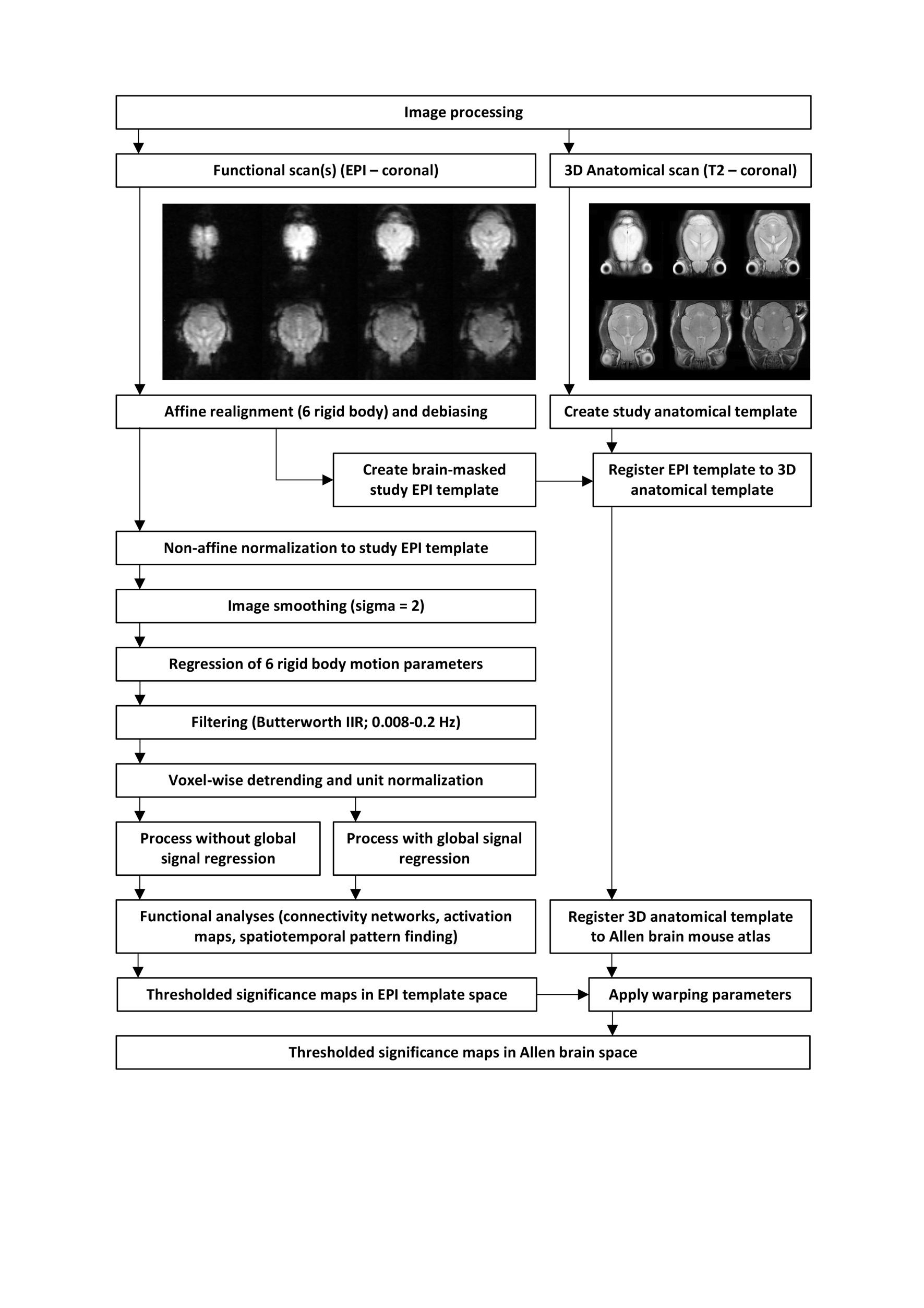
**

**Figure S2. Overview of image processing pipeline.** Coronal acquisition refers to the scan acquisition orientation in the scanner, but in fact corresponds to axial slices due to the animals’ prone position within the scanner.

**
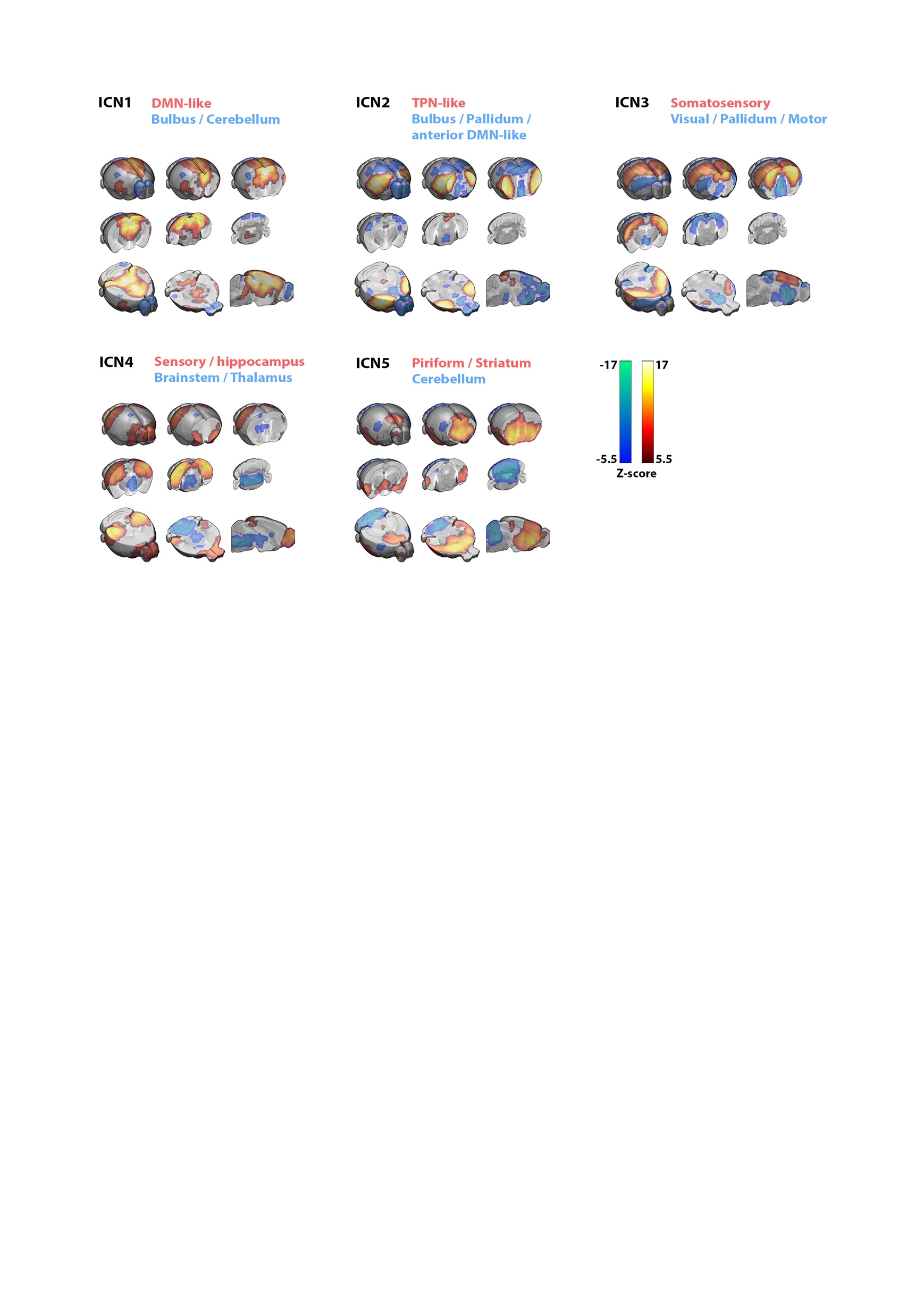
**

**Figure S3. Intrinsic connectivity networks.** Five major intrinsic connectivity networks (ICN) were observed during the resting state, displaying wide-spread anti-correlation. n = 71 scans. Maps display Z-scores (first level GLM; second level one sample T-test; T-scores normalized to Z-scores; FDR p<10^-7^).

**
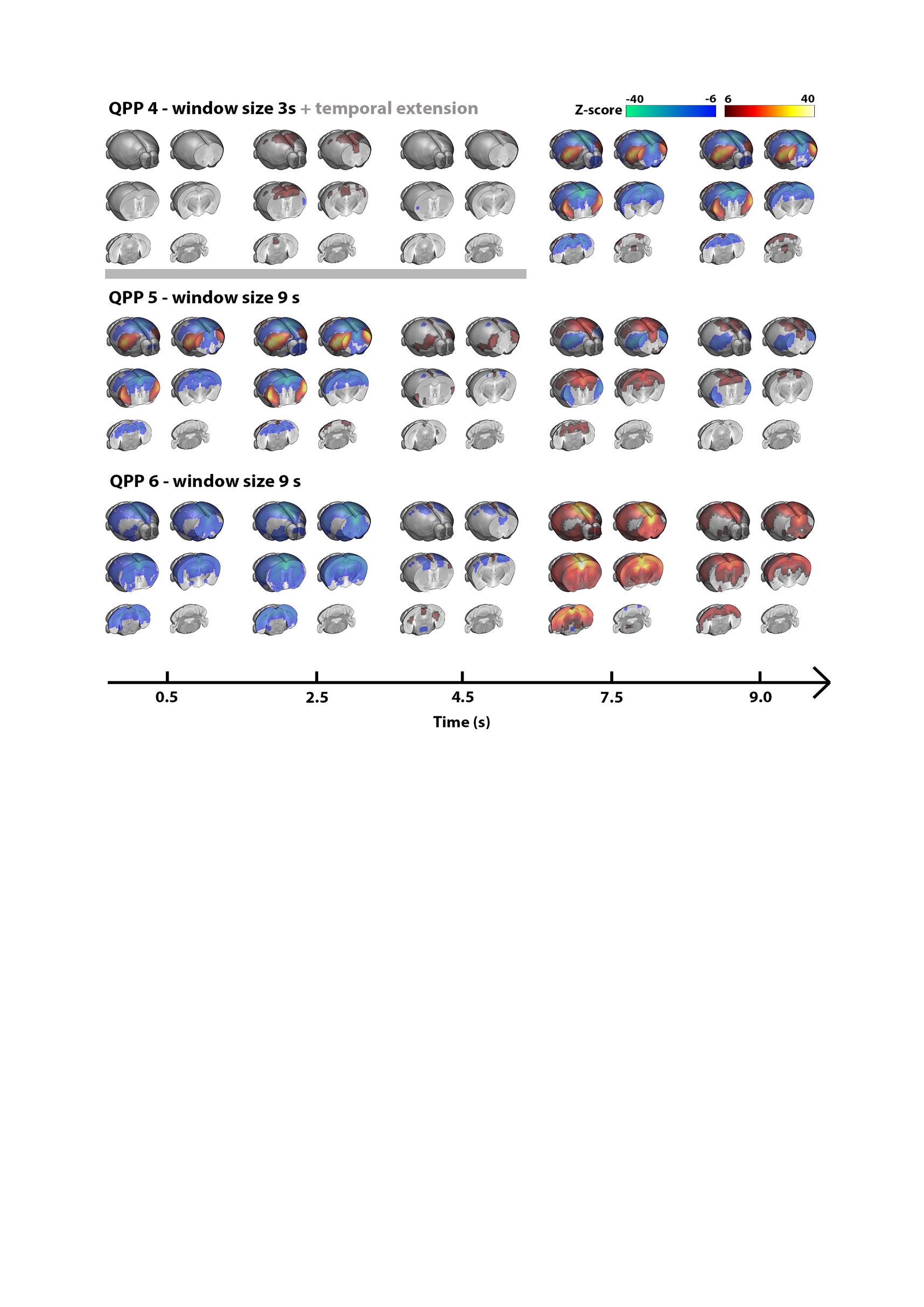
**

**Figure S4. Opposite phase QPPs.** This figure complements Figure 1 in the main text and shows the opposite phase variants of QPP1-3. QPPs are displayed on the same time axis (alignment through cross-correlation of QPP correlation vectors). Additional images before the 3s core of QPP4 were averaged to visualise likelihood of preceding activations. Maps display Z-scores [Z-test with H0 through randomized image averaging (n=1000), FDR p<10^-7^].


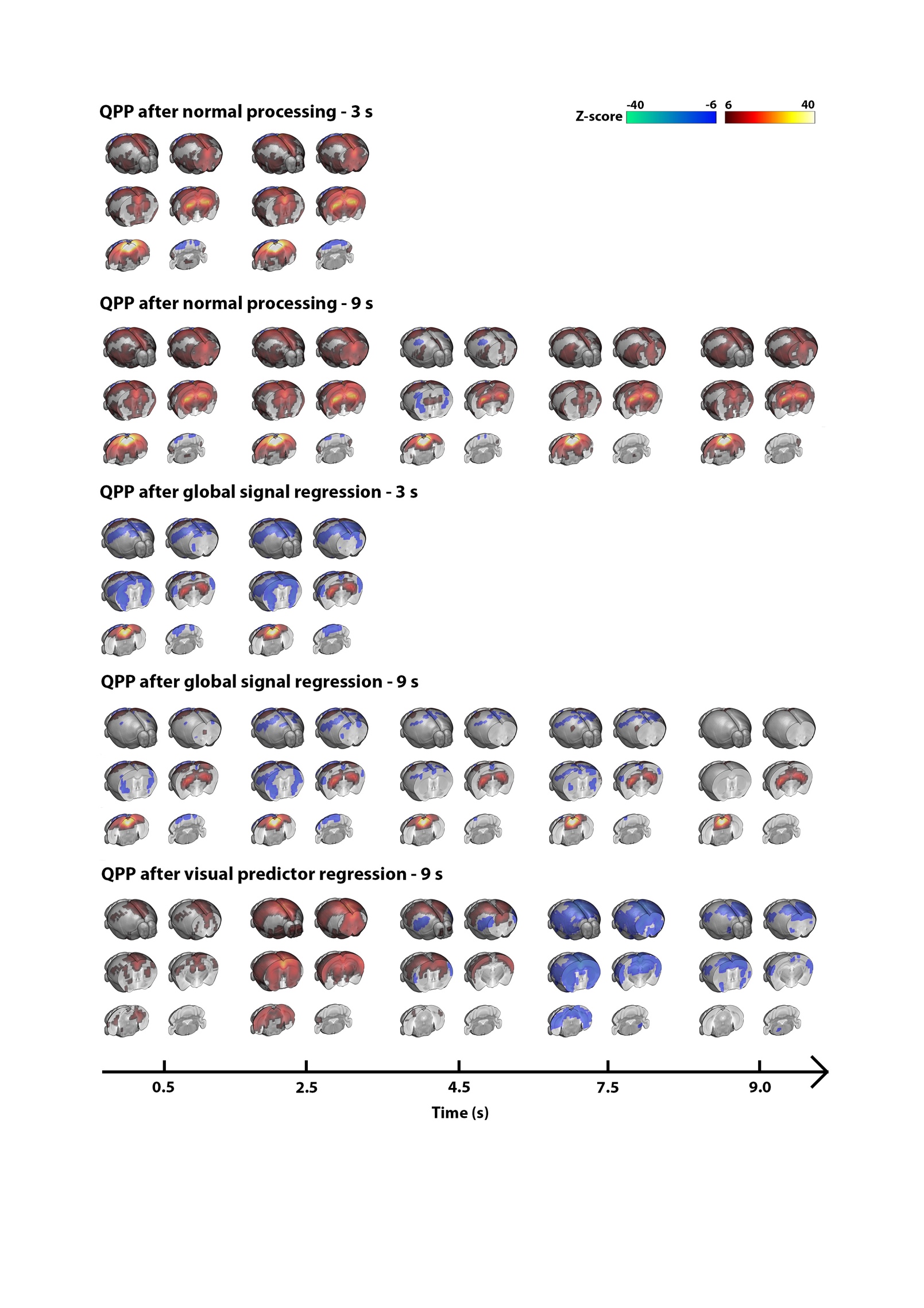


**Figure S5. QPPs during visual stimulation.** This figure complements Figure 2 in the main text and shows additional QPPs that could be determined from visual fMRI scans, using various regression methods (indicated atop respective images). The lowest shown 9s QPP, obtained after visual predictor regression, was similar to QPP3 in the resting state. N = 24 scans. Maps display Z-scores [Z-test with H0 through randomized image averaging (n=1000), FDR p<10^-5^].


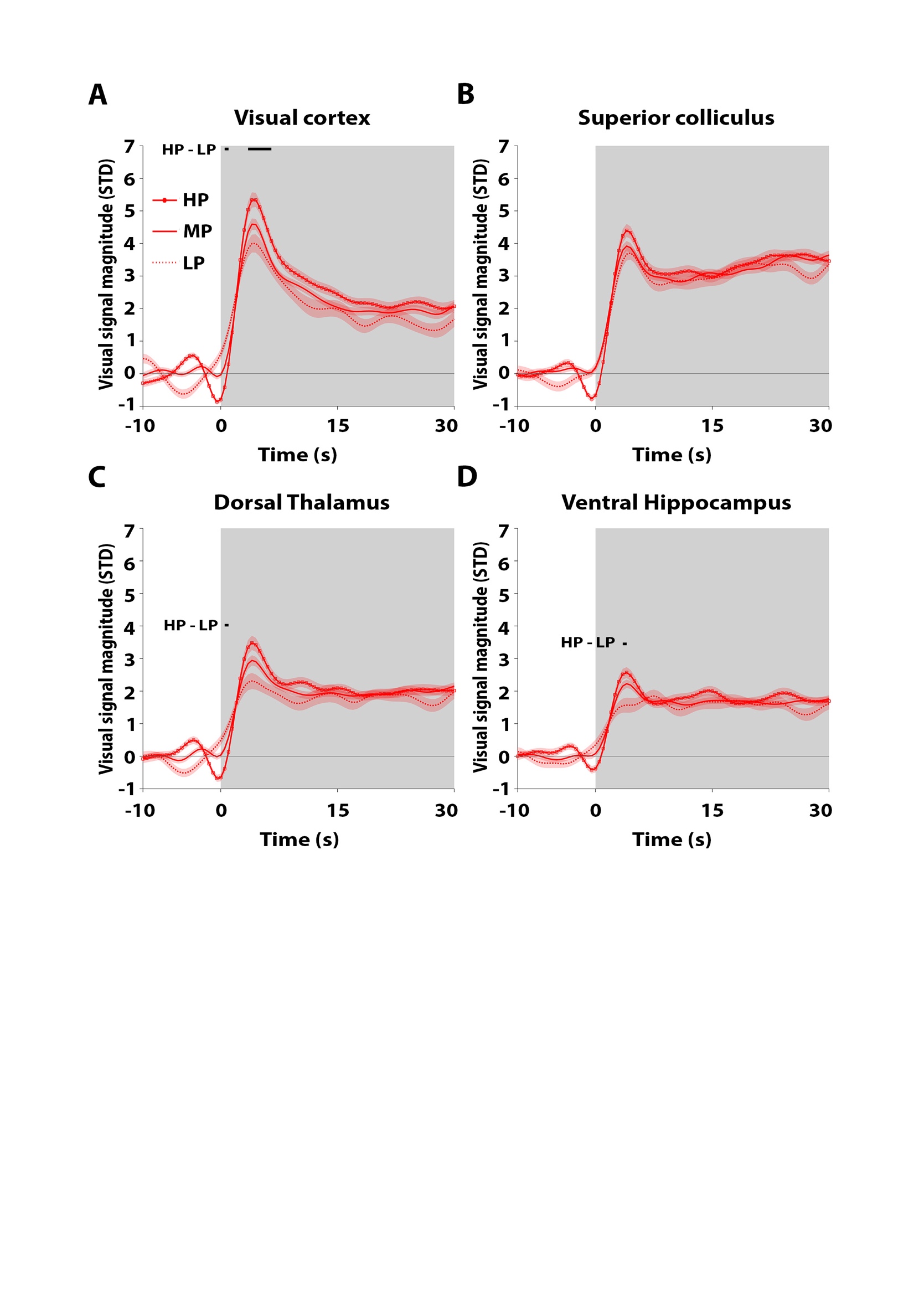


**Figure S6. QPP1-phase based stratification of visual stimulation trials broken down across brain areas.** This figure complements Figure 2 in the main text. The phase of QPP1 just prior to stimulation was used to stratify trials into three groups, depending on whether the phase was high (HP; [${}/4$,$3/4$]; i.e. ”peaks”), low (LP; [-3${}/4$,-${}/4$]; i.e. “valleys”), or medium (MP; [3${}/4$,-$3/4$] & [-${}/4$,${}/4$]; i.e. “slopes”). This highlighted significant stratification of visual signal amplitudes at transient responses in visual cortex and ventral hippocampus.

**A-D)** n = 24 scans in 24 mice. Grey areas mark trials (ON periods), traces show mean, patches show STE. Time traces are demeaned and variance normalized to 10s OFF period prior to stimulation. Black bars indicate significant differences between trial groups (One-way ANOVA, FDR (#bins) p<0.05; post hoc Bonferroni correction).

**
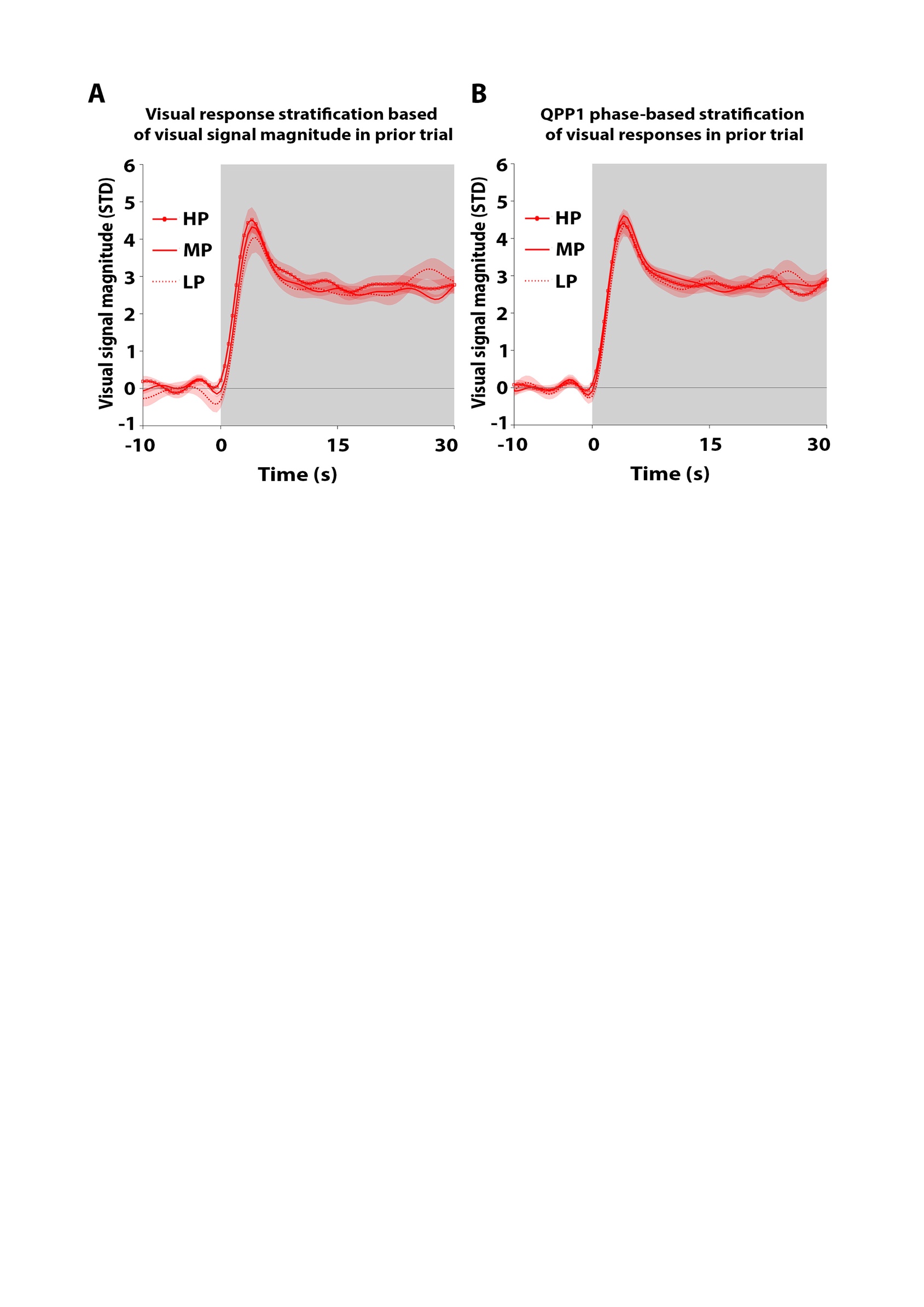
**

**Figure S7. Stratification of visual stimulation trials based off visual activations in the prior trials or QPP1 phase in the subsequent trial.** This figure complements Figure 2 in the main text. **A)** The magnitude of the visual signal (at the early transient response peak) in the preceding trial was used to stratify subsequent trials into three groups, respectively depending on whether the prior visual signal was high (HP; > 1 STD off all prior magnitudes), low (LP; < -1 STD off all prior magnitudes), or medium (MP; [-1 STD, 1 STD]). No significant differences were observed. This observation was consistent when using STD thresholds of 0.5 and 2.0 respectively (data not shown). **B)** The phase of QPP1 just prior to stimulation at a given trial was used to stratify preceding trials into three groups, respectively depending on whether the phase was high (HP; [${}/4$,$3/4$]; i.e. ”peaks”), low (LP; [-3${}/4$,-${}/4$]; i.e. “valleys”), or medium (MP; [3${}/4$,-$3/4$] & [-${}/4$,${}/4$]; i.e. “slopes”). No significant differences were observed. Note that this analysis is essentially identical to the one presented in Figure 2D, but now instead the QPP phase is used to stratify preceding trials.

**A-B)** n = 24 scans in 24 mice. Grey areas mark trials (ON periods), traces show mean, patches show STE. Time traces are demeaned and variance normalized to 10s OFF period prior to stimulation. Group differences were evaluated using One-way ANOVA (FDR (#bins) p<0.05; post hoc Bonferroni correction).

**
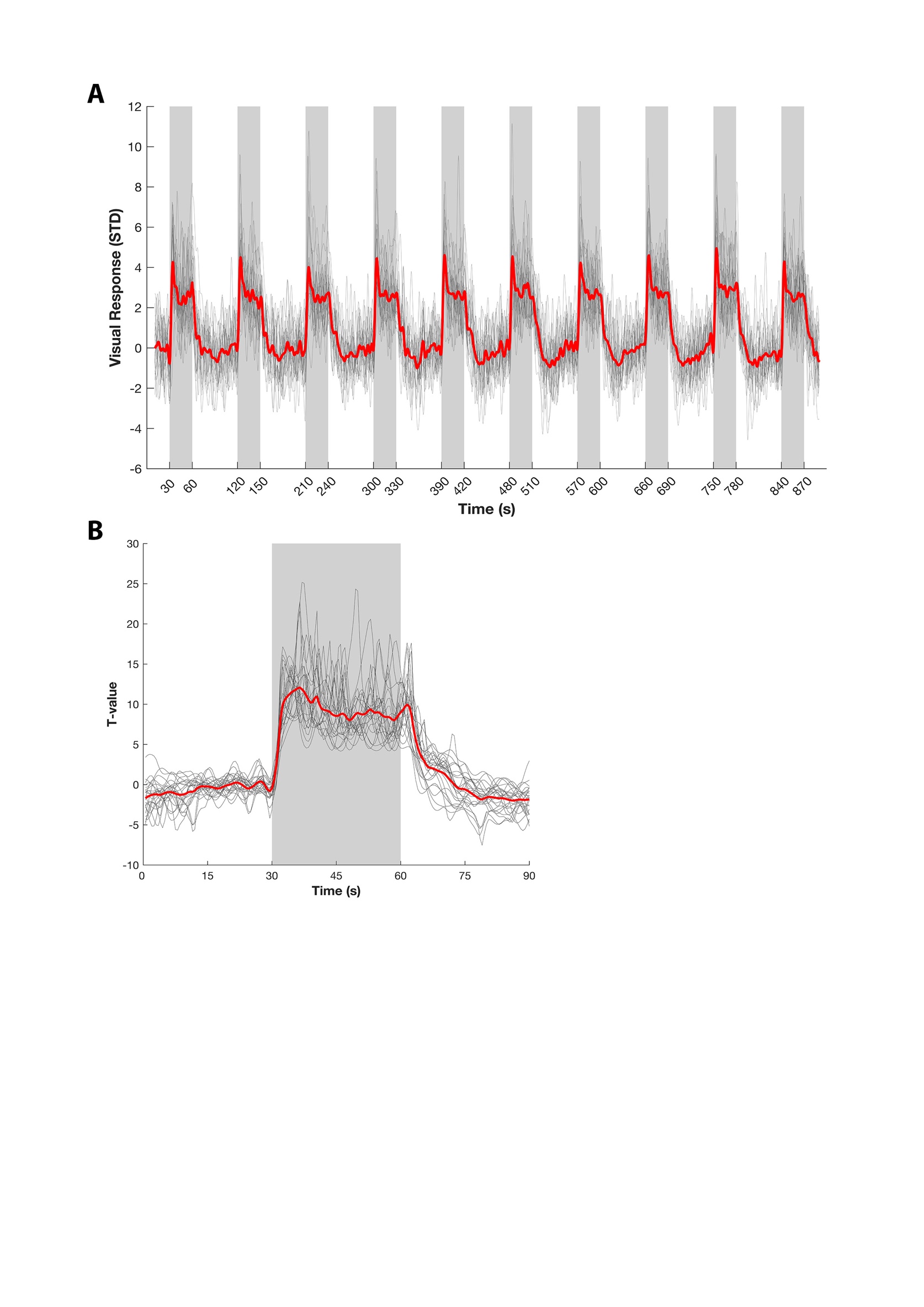
**

**Figure S8. Distribution of visual responses across animals and trials.** Visual responses across animals were highly consistent across consecutive trials **(A)**. The distribution of responses across trials (indicated through T-values) were also highly consistent across animals **(B)**.

**A-B)** Grey areas mark trials (ON periods), red traces show mean across animals, grey traces shown individual animals. Time traces are demeaned and variance normalized to 10s OFF period prior to stimulation. **B)** T-values were calculated at each time point of the 90s trial across 10 trials for a respective animal, providing a sense for the distribution of responses.

**
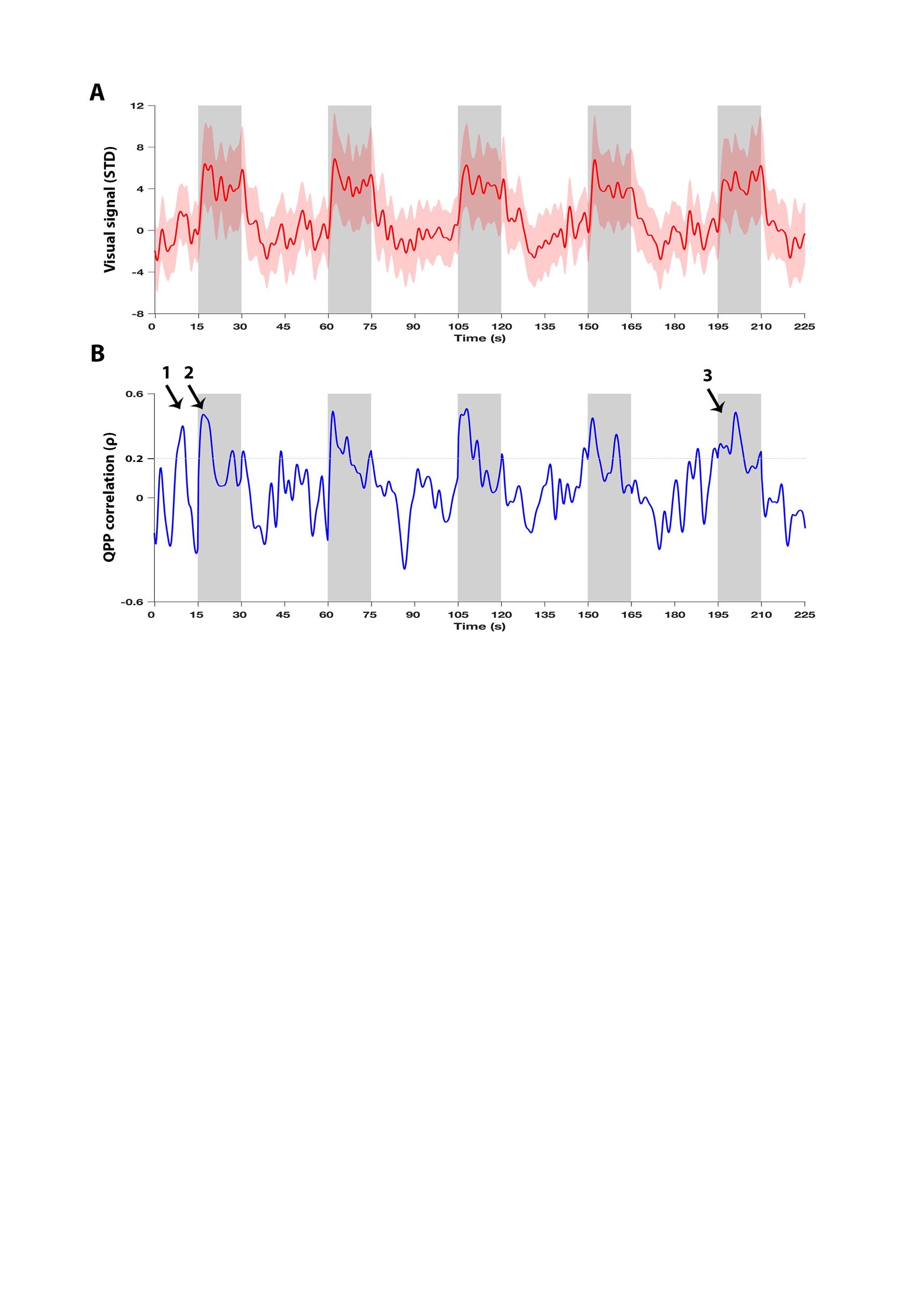
Figure S9. Single subject time courses during visual-evoked fMRI.** A 7-minute excerpt is shown from the visual-stimulation fMRI recordings in a single mouse (5 trials). Red trace shows mean signal from visual stimulus-evoked areas and patches show standard deviation across included voxels **(A)**. The blue trace shows the QPP1 correlation vector **(B)**. The dotted line in the lower panel (B) marks the correlation value of 0.2, which in the spatiotemporal pattern finding algorithm serves as a heuristic threshold for identifying QPP peak occurrences. This threshold may here be used as a suggestive reference to evaluate variance on the QPP correlation time series (blue trace). Black arrows indicate noteworthy observations: 1) strong QPP correlation peak during the period prior to a stimulation (OFF); 2) Strong QPP correlation peak at the start of stimulation (ON); 3) No QPP correlation peak at the start of stimulation (ON).

**
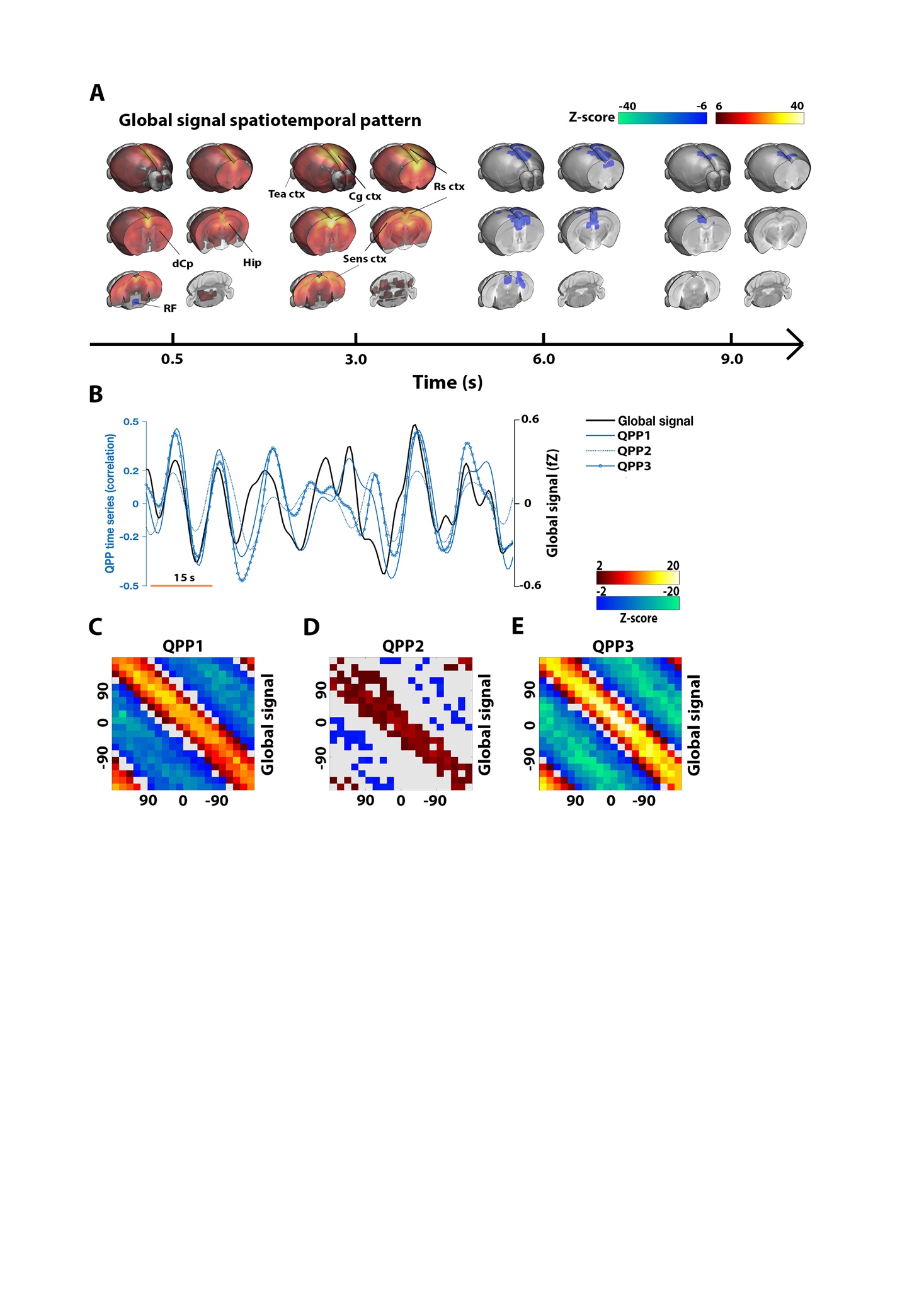
**

**Figure S10. Quasi-periodic brain patterns temporally coincide with global brain fluctuations.** The global signal was marked by a first phase of widespread activation, with stronger activations in sensory cortex and DMN-like areas **(A)**. A focal deactivation was also observed in the dorsal brain stem, at the height of the reticular formation. The second phase of the global signal mostly incorporated deactivation in Rs ctx and Cg ctx areas. Both visually **(B)** and based of phase-phase plots **(C-E)**, the global signal displayed clear temporal co-linearity with the three observed QPPs, with decreasing strength from QPP3 to QPP1 to QPP2.

**A-E)** n = 71 scans. **A)** Maps display Z-scores [Z-test with H0 through randomized image averaging (n=1000), FDR p<10^-7^]. **B)** Single subject excerpt. QPP correlation vectors represent Pearson correlations of QPPs with functional image series. Global signal fluctuations are shown as fisher Z-transformed intensities. Time series were aligned through cross-correlation (global signal peak occurred on average 2s into QPP1-3). **C-E)** Phase-phase plots show Z-scores [first level Z-test with H0 through randomized circular shuffling (n=1000); second level Z-test, FDR p<0.05].


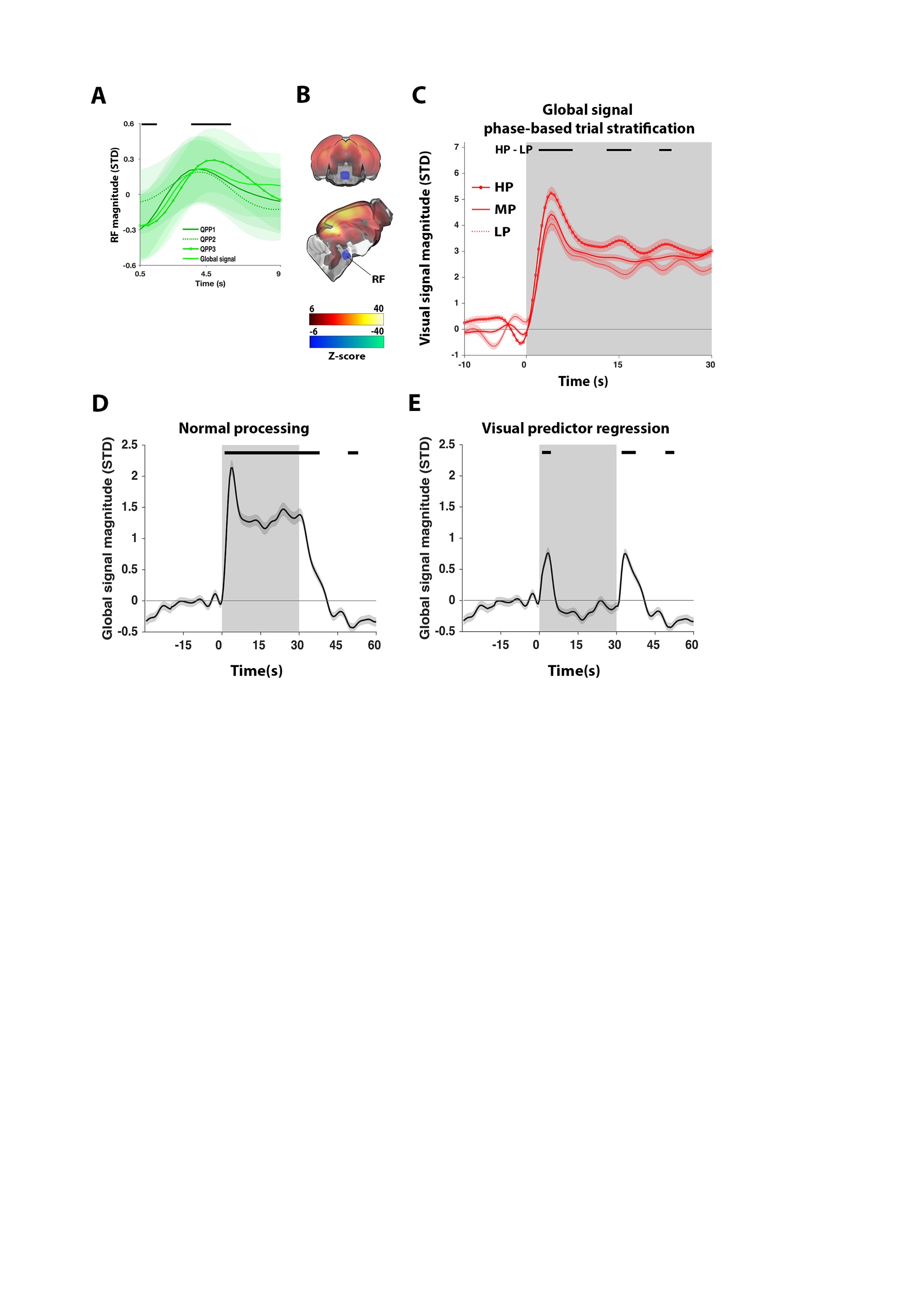


**Figure S11. The Global signal displays consistent properties compared to QPPs.** The global signal displayed significant de-activations in a focal dorsal brain stem area at the height of the RF, similar as what was observed for QPPs (image shows single a time point in the global signal spatiotemporal pattern, at time 0.5s, cfr. (B)). Further, time courses of the RF were on average highly similar across investigated QPPs and the global signal **(B)**. The phase of the global signal prior to stimulation could also be used to stratify stimulation trials into three groups and to show significant visual response differences at the start and during additional time periods of the stimulation block **(C)**. Finally, the global signal also displayed transient response properties to visual stimulation that were comparable to QPP1 and QPP3 **(D-E)**.

**A)** n = 71 scans. Maps display Z-scores [Z-test with H0 through randomized image averaging (n=1000), FDR p<10^-7^, cluster-correction 4 voxels]. **B)** n = 71 scans. Average RF time series across respective QPP correlation or global signal peaks (traces show mean; patches show STE). Black bars mark significant deviation from zero [statistical test as in (A)]. **C-E)** Grey areas mark trials (ON periods), traces show mean, patches show STE. Time traces are demeaned and variance normalized to 10s OFF period prior to stimulation. **C)** Black bars indicate significant differences between trial groups (One-way ANOVA, FDR (#bins) p<0.05; post hoc Bonferroni correction). **D-E)** Global signal time series (black) are respectively averaged across all trials and animals (n = 10 trials x 24 animals). Black bars mark significance (one sample T-test, FDR p<10^-5^).
