# Supplementary material for: Resting Brain Fluctuations Are Intrinsically Coupled to Visual Response Dynamics
Source: Cereb Cortex. 2020 Oct 27;31(3):1511–22. doi: 10.1093/cercor/bhaa305 (PMC7869084; doi:10.1093/cercor/bhaa305)
Supplement: Video_captions_bhaa305 [file video_captions_bhaa305.docx]

**Video captions**

**Video 1.** QPP1 temporal evolution displayed per TR (0.5s) over a duration of 3s. Maps display Z-scores [n = 71 scans; Z-test with H0 through randomized image averaging (n=1000), FDR p<10^-7^].

**Video 2.** QPP2 temporal evolution displayed per TR (0.5s) over a duration of 9s. Maps display Z-scores [n = 71 scans; Z-test with H0 through randomized image averaging (n=1000), FDR p<10^-7^].

**Video 3.** QPP3 temporal evolution displayed per TR (0.5s) over a duration of 9s. Maps display Z-scores [n = 71 scans; Z-test with H0 through randomized image averaging (n=1000), FDR p<10^-7^].
